# Heart rate variability, ECG minor changes, and HRV–ECG coupling associated with an adjunctive structured breathing–meditation protocol in combat-exposed military personnel

**DOI:** 10.3389/fnetp.2026.1912231

**Published:** 2026-08-13

**Authors:** Viktor S. Matsyshyn, Anatolii M. Kravchenko, Nataliia M. Ovodiuk, Dmytro Y. Zharkov, Kostiantyn O. Apykhtin, Oleksandr P. Romanchuk, Illya A. Chaikovsky

**Affiliations:** 1 State Administrative Department, State Institution of Science “Center of Innovative Healthcare Technologies”, Kyiv, Ukraine; 2 Department of Contactless Control Systems, V.M. Glushkov Institute of Cybernetics of the National Academy of Sciences of Ukraine, Kyiv, Ukraine; 3 Department of Vascular and Neuro-Cognitive Brain Diseases, D.F. Chebotarev Institute of Gerontology of the NAMS of Ukraine, Kyiv, Ukraine; 4 Department of Internal and Family Medicine, Lesya Ukrainka Volyn National University, Lutsk, Ukraine; 5 Department of Therapy and Rehabilitation, Ivan Bobersky Lviv State University of Physical Culture, Lviv, Ukraine

**Keywords:** technique of mental relaxation (TMR), post-traumatic stress disorder, military personnel, arterial hypertension, cardiovascular disease risk, electrocardiogram, heart rate variability, network physiology

## Abstract

**Introduction:**

Combat-related stress is associated with autonomic nervous system (ANS) dysregulation—sympathetic predominance, reduced vagal tone, and decreased heart rate variability (HRV)—linked to increased cardiovascular risk. Non-pharmacological interventions targeting autonomic regulation show promise, but controlled studies in combat-exposed populations remain limited. We assessed whether adding a structured breathing–meditation protocol, the Technique of Mental Relaxation (TMR), to standard rehabilitation is associated with changes in HRV and electrocardiogram (ECG)-derived indices of autonomic regulation in Ukrainian servicemen.

**Methods:**

A controlled, non-randomized study screened 84 male servicemen; 77 were allocated to a TMR group (n = 37; TMR plus standard treatment) or a control group (n = 40; standard treatment alone). Paired baseline and post-treatment 5-min supine recordings, obtained over a 14-day rehabilitation period, were available for 74 participants (TMR n = 35, control n = 39); a civilian reference group (n = 89) provided baseline comparison. Primary HRV outcomes were SDNN, RMSSD, the Baevsky Stress Index, and heart rate; ECG analysis added repolarization intervals, with Hedges’ g.

**Results:**

At baseline, both military groups showed impaired autonomic regulation versus civilians (all p < 0.0001). After intervention, the TMR group improved (SDNN 23→30 ms, p < 0.001; RMSSD 12→19 ms, p < 0.001; Stress Index 422→253, p < 0.001), whereas controls deteriorated; post-treatment between-group differences were significant for all primary outcomes (p ≤ 0.008).

**Conclusion:**

Heart-rate-adjusted analyses attenuated part of this difference, the Stress Index remaining most robust. Given the non-randomized design, these findings warrant caution and support randomized trials, with HRV as an objective monitoring tool.

## 1 Introduction

Armed conflicts impose substantial psychological and physiological burdens on military personnel, often leading to persistent stress-related disorders and impaired autonomic regulation. Chronic exposure to combat-related stress is associated with sustained activation of both the sympatho-adrenal-medullary (SAM) axis and the hypothalamic–pituitary–adrenal (HPA) axis, resulting in sympathetic predominance and reduced vagally mediated cardiac modulation. This imbalance contributes to autonomic nervous system (ANS) dysregulation, which manifests clinically as increased resting heart rate, reduced heart rate variability (HRV), sleep disturbances, emotional instability, and increased cardiovascular risk (Thayer et al., 2012; Kim et al., 2018; Shah et al., 2013; Romanchuk, 2023; Romanchuk, 2024).

Heart rate variability represents a widely recognized, non-invasive biomarker of ANS function and stress resilience. Reduced HRV, a physiological marker of autonomic dysregulation rather than a clinical manifestation in itself, has been consistently associated with post-traumatic stress disorder (PTSD), anxiety disorders, depression, and chronic stress conditions (Chalmers et al., 2014; Hauschildt et al., 2011). In military populations, decreased HRV has been reported as an objective indicator of combat-related psychophysiological strain and impaired adaptive capacity. Lower values of SDNN (standard deviation of all NN intervals) and RMSSD (root mean square of successive differences), along with elevated stress index parameters, have been observed among combat-exposed servicemen and correlated with severity of psychological symptoms and functional impairment (Matsyshyn et al., 2023). These findings highlight HRV as a potentially valuable tool for both diagnostic assessment and monitoring of rehabilitation outcomes in military populations.

Given the limitations of pharmacological approaches in managing chronic stress-related autonomic dysregulation, increasing attention has been directed toward non-pharmacological interventions aimed at restoring autonomic balance. Slow-paced breathing (SPB) at approximately six breaths per minute (0.1 Hz) has emerged as one of the most physiologically effective techniques for enhancing vagally mediated cardiac modulation. This breathing frequency corresponds to the resonance frequency of the cardiovascular system, maximizing respiratory sinus arrhythmia (RSA) and baroreflex sensitivity (BRS). These mechanisms enhance vagally mediated cardiac modulation and improve autonomic regulation (Lehrer and Gevirtz, 2014; Russo et al., 2017; Brown and Gerbarg, 2017).

A systematic review by Zaccaro et al. (2018) demonstrated that slow breathing practices produce multiple physiological and psychological effects, including increased parasympathetic activity, improved baroreflex function, enhanced alpha EEG activity, and reduced subjective stress levels. Similarly, a meta-analysis by Fincham et al. (2023) of 12 RCTs reported significant reductions in perceived stress (g = −0.35; p = 0.0009), anxiety (g = −0.32; p < 0.0001), and depressive symptoms (g = −0.40; p < 0.0001) following structured breathing interventions.

Meditation-based interventions have also demonstrated beneficial effects on autonomic regulation. A meta-analysis by Brown et al. (2021) found that mindfulness and meditation interventions increase vagally mediated HRV. Neurophysiological models suggest that meditation may enhance top-down regulation of autonomic function through prefrontal cortical networks and limbic system modulation (Thayer and Lane, 2009). Therefore, combining slow breathing with meditation may provide synergistic effects by targeting both peripheral autonomic mechanisms (baroreflex resonance, vagal afferent stimulation via Hering–Breuer reflex) and central regulatory pathways (prefrontal cortex → amygdala inhibition, neurovisceral integration).

These regulatory processes can be framed within Network Physiology, an emerging field that examines how diverse physiological systems and sub-systems dynamically interact, couple, and integrate as a coordinated network whose topology and coupling strength are hallmarks of physiological state and function (Bashan et al., 2012; Ivanov, 2021). From this perspective, heart rate variability (HRV) indexes the output of the autonomic control network, whereas electrocardiographic (ECG) repolarization morphology reflects the state of the cardiac electrophysiological sub-system; the coupling between these domains provides a multiscale, system-level readout of cardiovascular integration that spans beat-to-beat neural modulation, ∼0.1 Hz baroreflex oscillations, and slower rehabilitation-related adaptation (Bartsch et al., 2015). Combat-related stress may impair not only the function of individual regulatory nodes—expressed as reduced HRV—but also the dynamic coupling between the autonomic and cardiac sub-systems. Slow-paced breathing, by imposing a shared oscillatory drive at the cardiovascular resonance frequency, offers a physiologically plausible route to re-establishing such inter-system coordination. Accordingly, in addition to conventional HRV and ECG indices, the present study quantified HRV–ECG coupling as a network-physiological marker of autonomic–cardiac integration.

The ongoing armed conflict in Ukraine has created an urgent need for effective rehabilitation strategies for military personnel exposed to prolonged combat stress. In this context, the present study examined a structured breathing–meditation protocol referred to as the Technique of Mental Relaxation (TMR), which was operationalized as a standardized multi-component intervention integrating: (1) brief physical warm-up for somatic deactivation; (2) diaphragmatic breathing training; (3) slow-paced breathing (5 s inhalation, 5 s exhalation, without inter-breath pauses, ≈6 cycles/min), paced by instructor voice guidance together with a fixed auditory cue to maintain the target rhythm; and (4) observational meditation—non-judgmental awareness of breathing sensations. This multi-component structure targets vagal pathways, baroreflex sensitivity, and central autonomic regulation through mechanisms described in the literature (Lehrer and Gevirtz, 2014; Zaccaro et al., 2018).

Despite growing evidence supporting breathing and meditation interventions in civilian populations, controlled studies evaluating structured breathing–meditation protocols in combat-exposed military personnel remain limited. The present study aimed to assess HRV and ECG changes associated with adding a structured breathing–meditation protocol, referred to as TMR, to standard rehabilitation in combat-exposed military personnel. We hypothesized that participants receiving the adjunctive structured breathing–meditation protocol would demonstrate a more favorable autonomic trajectory than those receiving standard rehabilitation alone.

## 2 Materials and methods

### 2.1 Study design and ethical approval

This controlled clinical study was conducted at the State Institution “Center of Mental Health and Rehabilitation of Veterans ‘Lisova Polyana’” (Ministry of Health of Ukraine) during 2024. The study protocol was approved by the institutional ethics committee and conducted in accordance with the Declaration of Helsinki. All participants provided written informed consent.

### 2.2 Participants

**FIGURE 1:**
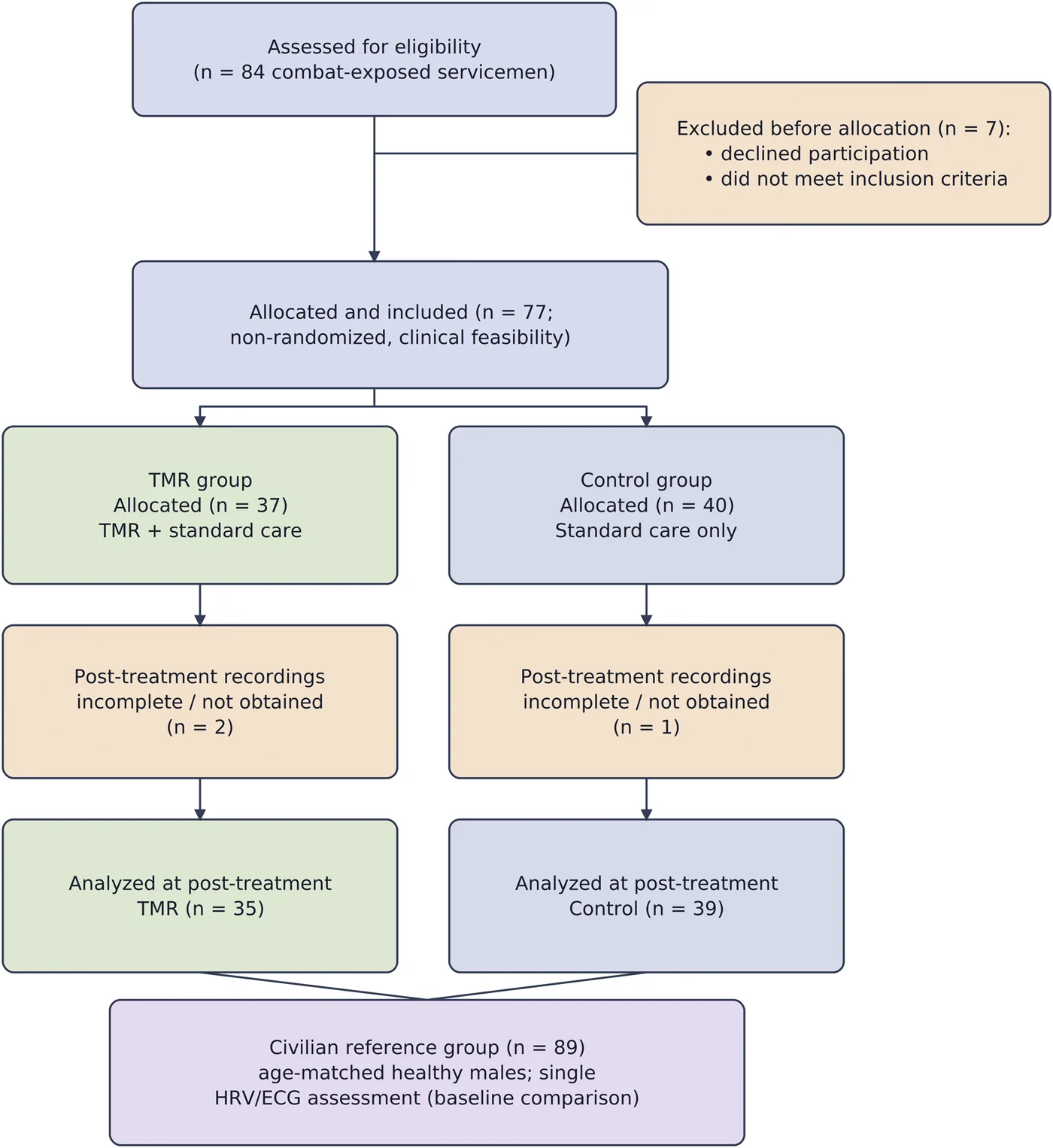
Participant flow (STROBE-style). Of 84 combat-exposed servicemen assessed for eligibility, 77 were included and allocated (non-randomized) to the TMR group (n = 37) or the control group (n = 40), with seven excluded before allocation (declined participation or did not meet inclusion criteria); valid post-treatment recordings were subsequently obtained for 35 and 39 participants, respectively; a civilian reference group (n = 89) provided normative comparison. ECG analysis sample sizes were reduced by signal-quality exclusions.

The study screened 84 male servicemen of the Armed Forces of Ukraine; 77 were allocated to the military study arms (TMR n = 37, Control n = 40) and had been exposed to active combat zones. A civilian reference group (n = 89) of age-matched healthy males without combat exposure was recruited for baseline HRV normative comparison (Figure 1).

#### 2.2.1 Clinical characteristics of participants

The military cohort (N = 84 screened, 77 allocated; valid paired baseline/post-treatment recordings available for 74 [TMR n = 35, Control n = 39]) comprised active-duty servicemen undergoing rehabilitation following combat exposure in eastern and southern Ukraine. The mean age was 44.9 ± 7.8 years (range 23–59) in the full allocated cohort (n = 77); in the subgroup with complete medical records used for Table 1 (n = 66), mean age was 47.6 ± 5.9 years in the TMR group and 45.7 ± 7.7 years in the control group. The average hospitalization duration was 23.6 ± 5.4 days. The predominant primary diagnoses included PTSD (F43.1; 55% of patients), adjustment disorders (F43.2; 49%), organic central nervous system (CNS) disorders following traumatic brain injury (TBI) (F07. x; 44%), and consequences of blast injury (T90.5; 29%). Multiple diagnoses were common, reflecting the polysyndromic nature of combat-related neuropsychiatric pathology. Types of combat exposure included blast injury/mine-explosive trauma with concussion and/or acoustic trauma in approximately 60% of cases, psychological trauma from direct combat participation, and combined mechanisms. Time from the primary traumatic event to hospitalization ranged from 1 to 120 months (group medians 13 and 19 months for the TMR and control groups, respectively; Table 1). Prior hospitalizations were documented in the majority of participants (>65%), indicating chronic or recurrent course of symptoms.

**TABLE 1:** Group-specific prevalence of principal comorbidities and additional baseline characteristics, based on structured chart review (n = 66 with complete medical records). p-values are two-sided Fisher’s exact test (categorical rows). None reached significance at α = 0.05, consistent with broadly comparable baseline comorbidity burden between groups, although the table was not powered for formal between-group inference.

Characteristic	TMR (n = 33)	Control (n = 33)	p (Fisher)
Arterial hypertension (any grade)	8 (24%)	13 (39%)	0.290
Hypertension grade II–III (severe)	3 (9%)	7 (21%)	0.303
Hypercholesterolemia (documented)	5 (15%)	8 (24%)	0.537
Degenerative spine disease	10 (30%)	10 (30%)	1.000
Any documented prior hospitalization	23 (70%)	24 (73%)	1.000
Age, years (mean ± SD)	47.6 ± 5.9	45.7 ± 7.7	—
Time from trauma to hospitalization, months, median [range]^a^	13 [1–120] (n = 19)	19 [1–96] (n = 16)	—

^a^ Time-to-hospitalization interval was explicitly documented in the medical record for a subset of participants (19/33 TMR, 16/33 Control); values are not available for the remainder and are not imputed. Body mass index, smoking status, and office blood pressure were available from the clinical registry for most participants and are reported by group in Supplementary Table S1; they were not incorporated into this table. Specific echocardiographic diagnoses (hypertensive heart disease, myocardial fibrosis, mitral/tricuspid valve insufficiency, diastolic dysfunction) were documented in narrative clinical records but were not consistently coded as structured, group-attributable variables across all charts; they are therefore described qualitatively at the cohort level only and are not broken out by group in this table.

#### 2.2.2 Comorbid conditions

Somatic comorbidities were prevalent and included arterial hypertension (24% of the TMR group and 39% of the control group; Table 1), degenerative spinal disease with chronic pain syndromes (30% in both groups; Table 1), chronic sensorineural hearing loss following acoustic trauma, gastrointestinal conditions (chronic gastritis, pancreatitis), urological conditions (chronic prostatitis, nephrolithiasis), and metabolic disorders (hypercholesterolemia, diabetes mellitus type 2 in isolated cases). Cardiovascular comorbidity included hypertensive heart disease (stage I–II), myocardial fibrosis, mitral/tricuspid valve insufficiency (grade I), and diastolic dysfunction (type I) without clinically significant heart failure. Group-specific prevalence for the principal comorbidities, based on a structured chart review of the 66 participants with complete medical records (33 TMR, 33 Control), is given in Table 1 below. Additional baseline characteristics (smoking status, office blood pressure, and body-mass index) are reported by group in Supplementary Table S1.

#### 2.2.3 Psychological screening at baseline

Available psychological screening data (NSI [Neurobehavioral Symptom Inventory] and PCL-5 [PTSD Checklist for DSM-5]) documented a mean NSI score of 41.8 (range 4–81) and mean PCL-5 score of 38.4 (range 3–71) at admission. Elevated NSI (≥40) was observed in approximately 45% of assessed patients, and PCL-5 scores consistent with probable PTSD (≥33) were present in approximately 50%. Suicidal ideation was actively screened for and denied by the vast majority of participants; two patients had a history of prior suicidal behavior (one suicide attempt, one passive suicidal ideation), both identified and managed with appropriate psychiatric oversight.

#### 2.2.4 Pharmacotherapy

Concurrent pharmacotherapy reflected standard clinical practice for combat-related stress disorders and included: atypical antipsychotics/anxiolytics (quetiapine [Quetiron] in low doses of 25–200 mg at bedtime, prescribed in >70% of patients); GABAergic anticonvulsants (pregabalin 75–150 mg, approximately 55%); antidepressants (SSRIs: paroxetine, escitalopram, sertraline, fluvoxamine—approximately 40%; trazodone—approximately 15%; SNRIs: venlafaxine, duloxetine—isolated cases); mood stabilizers (valproate, approximately 20%); melatonin-based hypnotics (Vita-Melatonin, >50%); neuroprotective agents (citicoline, Vitaxon/B-Vitamins, betahistine); and antihypertensive medications in patients with documented hypertension. Available chart review did not indicate a major medication-class imbalance between groups; however, complete per-patient dose-level pharmacotherapy data were not available for a formal group-by-group statistical comparison, and residual medication-related confounding cannot be excluded (cohort-level proportions in Supplementary Table S2).

Inclusion criteria comprised: active-duty military status with documented combat exposure; clinical signs of neuropsychic strain (anxiety, sleep disturbances, adjustment disorder, or mild-to-moderate PTSD); age 23–61 years (broadened from an initial 35–60-year target based on clinical feasibility, as eligible participants outside this range were included at treating-physician discretion); and ability to participate in breathing and meditation sessions. Exclusion criteria included: severe psychiatric disorders requiring acute intervention; clinically significant cardiac arrhythmias or conduction blocks; chronic cardiovascular disease in decompensated stage; use of beta-adrenergic blockers; and refusal to participate.

### 2.3 Group allocation

Participants were allocated into two groups based on clinical and organizational considerations within the rehabilitation setting. Randomization was not performed. The TMR group (n = 37 allocated; n = 35 analyzed at post-treatment) received the Technique of Mental Relaxation in addition to standard treatment. The Control group (n = 40 allocated; n = 39 analyzed at post-treatment) received standard treatment only. This non-randomized design should be considered when interpreting the results, and the findings are presented as preliminary controlled evidence rather than definitive causal proof.

### 2.4 Intervention: technique of mental relaxation (TMR)

In the present study, the Technique of Mental Relaxation (TMR) was defined as a standardized structured breathing–meditation protocol comprising four sequential components: brief physical warm-up, diaphragmatic breathing, slow-paced breathing, and observational meditation. The intervention was used as an adjunct to standard rehabilitation and was not intended to replace pharmacological, psychological, or physiotherapeutic treatment. Each session was conducted twice daily (morning and evening), lasting 15–20 min, for approximately 14 days (corresponding to the standard rehabilitation duration; individual variation 10–14 days depending on the treatment schedule). The protocol consisted of four sequential stages:

Stage 1 — Physical warm-up (1–3 min): Gentle stretching and body scanning for initial somatic deactivation.
Stage 2 — Diaphragmatic breathing (2–3 min): Establishing abdominal breathing pattern with attention to respiratory mechanics.
Stage 3 — Slow-paced breathing (3–5 min): Inhale 5 s, exhale 5 s, without inter-breath pauses, approximating 0.1 Hz (6 cycles/min). This frequency is thought to engage baroreflex resonance, maximize respiratory sinus arrhythmia, and enhance vagally mediated cardiac modulation.
Stage 4 — Observational meditation (5–10 min): Non-judgmental observation of spontaneous breathing without controlling respiratory rhythm. Participants were instructed to maintain relaxed attention and allow spontaneous thoughts without engagement.

Sessions were initially guided by a trained neurologist. Adherence was monitored through daily session logs. Participants in the Control group did not practice any structured breathing or meditation exercises during the study period.

### 2.5 Standard rehabilitation program

Both groups received standard rehabilitation including pharmacotherapy according to national clinical protocols (anxiolytics, hypnotics, or antidepressants as clinically indicated), psychotherapy sessions, physiotherapy (massage, therapeutic exercise, craniosacral therapy, biofeedback), and psychological rehabilitation.

### 2.6 HRV assessment

Five-minute resting ECG recordings were obtained using the “Oracul/Harmony” ECG-HRV analysis system (Ukraine) at a sampling rate of 500 Hz, in a supine position, under standardized conditions (quiet environment, spontaneous breathing, resting state, morning recording, minimum 2 h after physical exertion or caffeine). Measurements were performed at baseline (beginning of rehabilitation) and after the 14-day treatment course. RR-interval (RR) series underwent automated artifact detection and correction within the Harmony system, with visual verification of the tachogram prior to analysis; non-sinus beats and ectopic intervals were flagged and excluded from HRV computation. Recordings with more than 5% edited intervals were excluded from spectral analysis. Frequency-domain indices were derived over standard bands—very-low-frequency (VLF, 0.0033–0.04 Hz), low-frequency (LF, 0.04–0.15 Hz), and high-frequency (HF, 0.15–0.40 Hz) — following international standards (Task Force, 1996). Available signal-processing details, including frequency-band definitions, artifact handling, and the standardized stationarity-related recording conditions, are detailed in Supplementary Material S1, covering artifact detection and correction, interpolation and resampling, the spectral estimator with its window and segment settings, and detrending; R-peak detection is performed within the device firmware. The Baevsky Stress Index (SI) was computed as SI = AMo/(2 × Mo × ΔX) (Baevsky et al., 1984; Baevsky and Ivanov, 2001), where AMo is the amplitude of the mode (%), Mo the mode of the RR-interval distribution (s), and ΔX the variation range of RR intervals (s).

### 2.7 Electrocardiogram assessment

In addition to HRV analysis, ECG signals recorded in six limb leads (I, II, III, aVR, aVL, aVF) were analyzed using the Oracul/Harmony ECG-HRV analysis system. A broad set of ECG-derived parameters, including both conventional and software-derived exploratory indices, was calculated. The present analysis focused on: standard conduction intervals (PQ, QRS, QT); rate-corrected repolarization intervals (QTc by Bazett formula and QTcF by Fridericia formula); indices of repolarization morphology and spatial orientation of the T-vector (Tp–Te interval; QRS–T angle; alpha-T angle in the frontal plane); ST-segment deviations at J+80 ms; integral ST–T shape indices per lead; T-wave amplitude, symmetry and area indices; the Macruz index [P/(PQ−P)]; cardiac cycle proportionality indices including K1 = (PQ+QTc)/RR and a golden ratio index; and a functional heart failure signs index derived from ECG morphology (Chaikovsky, 2020). Software-derived ECG indices were considered exploratory; their algorithms and normative ranges are proprietary to the device manufacturer and are not publicly documented, and they are therefore reported for exploratory purposes only.

These parameters were analyzed at baseline and after the 14-day treatment period, in the same recording positions as HRV parameters (5-min supine). Because heart rate was derived from two signal sources, 2 HR values are reported: the HRV-derived HR was computed from normal-to-normal (NN) intervals of the rhythmogram used for HRV analysis, whereas the ECG-derived HR was obtained from QRS detection in the six-lead ECG subset. Unless otherwise stated, the ECG-derived HR is used for repolarization interpretation and the HRV-derived HR for autonomic interpretation.

### 2.8 Outcome measures

#### 2.8.1 Primary outcomes

Change in SDNN (standard deviation of NN intervals), RMSSD (root mean square of successive differences), Baevsky Stress Index (SI), and resting heart rate (HR). These parameters were selected *a priori* as established, widely validated markers of overall autonomic variability, vagally mediated short-term cardiac modulation, regulatory rigidity/sympathetic predominance, and cardiovascular arousal.

SI is interpreted as an index of sympathetic dominance, a state that may arise from parasympathetic withdrawal without a necessary increase in sympathetic activity (Baevsky and Ivanov, 2001).

#### 2.8.2 Secondary/exploratory outcomes

pNN20 and pNN50 (percentage of adjacent NN intervals differing by more than 20 ms and by more than 50 ms, respectively), SDSD (standard deviation of successive differences), approximate entropy, HF power (high-frequency spectral component), LF power, LF/HF ratio, detrended fluctuation analysis (DFA) exponent, triangular index, and software-derived composite indices of the “Oracul/Harmony” system: Operational Regulatory Control, Regulatory Reserve State, Complex Regulatory Index, Index of Vegetative Regulation (IVR), as well as McCraty psychoemotional tension grade (McCraty et al., 2009), and Baevsky Functional State classification (Baevsky and Ivanov, 2001). The composite indices represent averages of constituent parameters scaled to a 0–100 metric (Chaikovsky et al., 2026); their exact algorithms and normative ranges are proprietary to the device manufacturer and are not publicly documented.

ECG-derived outcomes were considered exploratory and included conventional repolarization intervals, repolarization morphology indices, T-wave amplitude and symmetry parameters, and software-derived cardiac cycle proportionality indices. These outcomes were analyzed to complement HRV findings and should not be interpreted as primary efficacy endpoints.

Resting HR (bpm) and arterial blood pressure (BP, mmHg by Korotkoff auscultation) were recorded at both time points.

### 2.9 Statistical analysis

Data processing was performed using Python (NumPy, SciPy, pandas) within a custom automated statistical pipeline. For each parameter, intra-group comparisons (pre vs. post) and inter-group comparisons were conducted using Student’s t-test (normally distributed data) or Wilcoxon signed-rank/Mann–Whitney U-test (non-normal distributions). Results are presented as medians with interquartile ranges (IQR) given the non-normal distribution of most HRV parameters. Effect sizes were quantified using Hedges’ g (corrected Cohen’s d for small-sample bias), with standard thresholds: |g| ≥ 0.20 (small), ≥0.50 (moderate), ≥0.80 (large), and ≥1.20 (very large). Statistical significance was set at p < 0.05.

Normality of distributions was assessed using the Shapiro–Wilk test. In response to reviewer feedback, we additionally computed rate-corrected HRV indices (RMSSD/RR and SDNN/RR, using RR = 60000/HR in ms) for the complete valid analysis sample with paired supine HR and HRV records traceable to source instrument files (33 TMR, 34 Control), to evaluate whether pre–post and between-group differences in RMSSD and SDNN persist after normalizing for the concurrent change in mean heart rate.

The global multivariate association between the HRV and ECG parameter blocks was quantified using the RV coefficient (Escoufier, 1973; Robert and Escoufier, 1976), with statistical significance assessed by permutation testing (5,000 permutations; Josse et al., 2008) and small-sample upward bias evaluated using the modified RV coefficient (Smilde et al., 2009) and the permutation-derived null expectation.

Because resting heart rate is a physiological determinant and a mathematical confounder of HRV rather than an HRV outcome in its own right (Sacha and Pluta, 2008; Sacha, 2014), the post-treatment between-group HRV comparison (TMR vs. Control) was additionally performed with adjustment for heart rate using rank-based analysis of covariance, an approach appropriate for the non-normal distribution of HRV parameters (Estévez et al., 2015).

Analyses beyond the predefined primary outcomes were exploratory and should be interpreted with caution due to the absence of correction for multiple comparisons. Between-group comparisons were performed using post-treatment values and, where data permitted, change scores (post−pre) to estimate differences in treatment response. Because baseline HRV values differed between groups, post-treatment comparisons should be interpreted cautiously; future studies should use ANCOVA-adjusted primary analyses to adequately control for baseline differences and regression to the mean.

To characterise the relationship between autonomic regulation indices and ECG morphological markers of myocardial state (HRV-ECG coupling), pairwise Spearman rank correlations were computed between all HRV parameters (25 indices) and all ST-T morphological ECG parameters (25 parameters covering the ST segment, T-wave form, and integral ST-T shape indices in six limb leads) for each group and time point separately. Multiple-comparison correction was applied using the Benjamini–Hochberg false discovery rate (FDR) procedure at q < 0.05 across all 625 pairwise tests per group. To assess whether significant associations were independent of heart rate, partial Spearman correlations were computed for the most prominent pairs after residualising both variables on HR. These analyses were exploratory and hypothesis-generating.

## 3 Results

### 3.1 Baseline: military groups vs. civilian reference

At baseline, both military groups demonstrated significantly impaired autonomic regulation compared with the civilian reference group, with statistically significant differences across most HRV parameters (Table 2). Military personnel showed substantially lower HRV (SDNN: 23–29 vs. 39 ms; RMSSD: 12–16 vs. 24 ms), elevated stress indices (SI: 347–422 vs. 187 units; IVR: 589–664 vs. 298 units), reduced entropy (0.22–0.29 vs. 0.45), and lower composite regulatory indices.

**TABLE 2:** Baseline HRV parameters in military groups vs. civilian reference (medians).

Parameter	Control (n = 40)	TMR (n = 37)	Civilian (n = 89)	Δ Ctrl vs. Civ	p	Δ TMR vs. Civ	p
	Median [IQR] (range)	Median [IQR] (range)	Median [IQR] (range)				
SDNN, ms*	29 [22–38] (8–60)	23 [17–28] (11–78)	39 [31–47] (15–91)	10	<0.0001	16	<0.0001
RMSSD, ms*	16 [14–25] (2–54)	12 [9–16] (4–77)	24 [17–31] (8–109)	8	<0.0001	12	<0.0001
SI, units*	347 [179–500] (60–1860)	422 [361–823] (40–1439)	187 [125–301] (26–771)	−160	<0.0001	−235	<0.0001
HR, bpm*	76.5 [66.0–81.0] (51.0–112.0)	78 [71.0–85.0] (57.0–100.0)	73 [67.0–79.0] (56.0–105.0)	−3.5	0.057	−5	0.013
pNN20, %	12.5 [10–32] (0–74)	6 [2–17] (0–81)	33 [19–50] (2–83)	20.5	<0.0001	27	<0.0001
SDSD, ms	10 [9–16] (2–31)	7 [6–10] (2–46)	15 [11–19] (5–85)	5	0.0003	8	<0.0001
Entropy^†^	0.29 [0.23–0.49] (0.00–0.83)	0.22 [0.09–0.42] (0.00–0.86)	0.45 [0.29–0.61] (0.02–0.85)	0.16	<0.0001	0.23	<0.0001
IVR, units	588.5 [293–756] (142–1965)	664 [584–1129] (82–1812)	298 [214–488] (53–1037)	−290.5	<0.0001	−366	<0.0001

Baseline (pre-treatment) values; 5-min supine recordings (civilian reference: single measurement). Values are medians with interquartile range [IQR] and (minimum–maximum). Between-group comparisons vs. civilians: Mann–Whitney U-test. Δ, difference in medians. SDNN, standard deviation of NN, intervals; RMSSD, root mean square of successive differences; SI, baevsky stress index; HR, heart rate; pNN20, proportion of successive NN, differences >20 ms; SDSD, standard deviation of successive differences; IVR, index of vegetative regulation.

^†^ approximate-entropy index (Harmony device). Baseline medians in this table are computed on the full allocated cohort (Control n = 40, TMR n, 37); the corresponding baseline medians in Tables 4, 6 are computed on the paired complete-case sample (Control n = 39, TMR n, 35) and therefore differ slightly, whereas interquartile ranges and ranges are essentially unchanged.

* Primary outcomes. Δ = civilian median − military-group median.

At baseline, both military groups showed several ECG differences from the civilian reference group rather than a clearly TMR-specific ECG phenotype (Table 3). T-wave amplitude in lead I was lower in both military groups compared with civilians, with the largest numerical reduction in the TMR group (TMR 155 μV, Control 204 μV, Civilian 309 μV; both p < 0.001 vs. civilians). The alpha-T angle in the frontal plane was wider in both military groups than in civilians (TMR 44°, Control 40°, vs. Civilian 22°; p = 0.003 and p = 0.004, respectively), and the integral ST–T index in lead aVL was lower in the TMR group versus civilians (41 vs. 75; p < 0.001), with a smaller, non-significant difference for the control group. The Tp–Te interval did not differ meaningfully between groups at baseline (0.082 s in all three groups; p = 0.32, non-significant). The myocardial state index was similar across groups (TMR 62, Control 63, Civilian 64; all pairwise comparisons non-significant). QTcF was modestly elevated in both military groups versus civilians (Control 0.425, TMR 0.424, vs. Civilian 0.412; p = 0.0004 and p = 0.002, respectively), though median values remained within accepted clinical limits (QTcF <0.450 s in men). These findings suggest stress-related repolarization differences in the military cohort as a whole, rather than a baseline ECG phenotype specific to the participants later assigned to TMR. Both groups showed significantly elevated integral ST–T shape indices in lead III compared with civilians, consistent with inferior-axis repolarization changes common in stress-related and sympathetically dominant states.

**TABLE 3:** Baseline ECG parameters in military groups vs. civilian reference.

Parameter	Control (n = 40) median [IQR] (range)	TMR (n = 37) median [IQR] (range)	Civilian (n = 89) median [IQR] (range)	Δ Ctrl vs. Civ	p	Δ TMR vs. Civ	p
HR, bpm	73.0 [66.0–81.0] (51.0–112.0)	77.0 [71.0–85.0] (57.0–100.0)	73.0 [67.0–79.0] (56.0–105.0)	+0	0.783	+4	0.103
QT interval, s	0.393 [0.374–0.416] (0.345–0.478)	0.392 [0.376–0.405] (0.320–0.453)	0.384 [0.367–0.400] (0.325–0.467)	+0.009	0.078	+0.008	0.136
QTc (Bazett), s	0.440 [0.428–0.448] (0.399–0.501)	0.443 [0.425–0.459] (0.398–0.493)	0.427 [0.416–0.439] (0.366–0.479)	+0.013	0.001	+0.016	0.002*
QTcF (Fridericia), s	0.425 [0.413–0.433] (0.380–0.493)	0.424 [0.411–0.436] (0.370–0.465)	0.412 [0.400–0.421] (0.357–0.475)	+0.013	<0.001*	+0.012	0.002*
Tp–Te interval, s	0.082 [0.078–0.092] (0.068–0.212)	0.082 [0.077–0.092] (0.066–0.148)	0.082 [0.078–0.086] (0.068–0.106)	+0	0.428	+0	0.316
QRS duration, s	0.102 [0.094–0.106] (0.066–0.130)	0.098 [0.092–0.106] (0.062–0.120)	0.102 [0.094–0.108] (0.080–0.128)	+0	0.980	−0.002	0.371
PQ interval, s	0.160 [0.151–0.182] (0.110–0.230)	0.156 [0.142–0.174] (0.110–0.190)	0.152 [0.140–0.166] (0.108–0.220)	+0.008	0.009*	+0.004	0.290
Macruz index	1.77 [1.23–2.36] (0.80–33.00)	1.75 [1.46–2.16] (0.37–7.70)	1.37 [1.02–2.00] (0.54–28.00)	+0.4	0.008*	+0.38	0.003*
Myocardial state index	63 [57–70] (39–81)	62 [54–65] (44–86)	64 [59–68] (43–77)	−1	0.990	−2	0.133
Integral ST–T index (lead II)	68.5 [51.2–77.2] (12.0–93.0)	66.0 [45.0–74.0] (0.1–96.0)	62.0 [44.0–74.0] (10.0–100.0)	+6.5	0.305	+4	0.824
Integral ST–T index (lead III)	23.5 [10.0–51.2] (10.0–75.0)	22.0 [10.0–40.0] (0.0–100.0)	11.0 [10.0–26.0] (10.0–93.0)	+12.5	0.005*	+11	0.012*
T-wave amplitude, lead I, µV	204 [140–280] (36–374)	155 [104–231] (0–467)	309 [221–387] (68–709)	−105	<0.001*	−154	<0.001*
T-wave amplitude, lead III, µV	45 [-44–113] (-176–296)	53 [-28–122] (-185–265)	−32 [-137–82] (-341–255)	+77	0.006*	+85	0.010*
Alpha-T angle, frontal, °	40 [18–54] (-3–83)	44 [20–57] (-39–68)	22 [7–41] (-40–76)	+18	0.004*	+22	0.003*
QRS–T angle, frontal, °	18 [10–33] (1–120)	14 [8–42] (0–103)	26 [16–47] (0–102)	−8	0.084	−12	0.059
HF signs index (ECG)	0.1 [0.1–0.2] (0.0–0.6)	0.18 [0.11–0.24] (0.06–0.39)	0.1 [0.1–0.2] (0.0–0.5)	+0	0.387	+0.1	0.043*

Baseline (pre-treatment) ECG, parameters; 5-min supine recordings (civilian reference: single measurement). Values are medians with interquartile range [IQR] and (minimum–maximum). Between-group comparisons vs. civilians: Mann–Whitney U-test. HR, heart rate; QTc, rate-corrected QT (Bazett); QTcF, rate-corrected QT (Fridericia); Tp–Te, peak-to-end of T wave; ST, dev., ST-segment deviation; ISTT, integral ST–T, shape index; K1, composite conduction–repolarization index. HR, values in this table are derived from ECG QRS, detection. The myocardial state index and Golden ratio index are proprietary Harmony device indices, not standard ECG, parameters; values reported for exploratory purposes. Group headers indicate the allocated baseline cohorts (TMR n = 37, Control n = 40); parameter-specific available counts are reported in Supplementary Table S3, with a maximum of 36 TMR, and 39 Control baseline ECG, recordings.

Parameters with p < 0.05 in either military group vs. civilian comparison (Mann–Whitney U test, * denotes p < 0.05). Δ = group median − Civilian median. QTc/QTcF civilian reference from age-matched normative data (Task Force, 1996). ns = not significant or civilian reference unavailable for this proprietary parameter. One TMR, baseline recording with an internally inconsistent derived-parameter export (QTcF, Tp–Te, QRS, duration, HF-signs index, and the Golden ratio index all implausible despite a normal QT/QTc, for the same beat) was excluded from these five derived indices only; the participant’s HR, QT, QTc, and all other parameters were retained. Excluding this record left the reported medians and IQR, materially unchanged and narrowed the reported ranges accordingly.

### 3.2 Baseline comparison between military groups

The two military groups were generally comparable at baseline for HR (TMR 78 vs. Control 76.5 bpm, p = 0.64), overall functional state, and psychological tension grade. However, the TMR group exhibited somewhat worse initial HRV: lower SDNN (23 vs. 29 ms), lower RMSSD (12 vs. 16 ms, p = 0.0003), and higher IVR (664 vs. 589, p = 0.003). These baseline differences indicate that the group subsequently receiving TMR had more pronounced autonomic dysregulation, which should be considered when interpreting the magnitude of post-treatment changes.

### 3.3 Intra-group changes

Tables 4, 5 present the primary and key secondary outcomes for both groups before and after the 14-day intervention period. Baseline values in Tables 4, 5 are computed on the paired complete-case sample (TMR n = 35, Control n = 39) and therefore differ slightly from the baseline medians in Tables 2, 3, which are based on the full allocated cohort (TMR n = 37, Control n = 40); interquartile ranges and ranges are essentially unchanged.

**TABLE 4:** HRV changes in TMR and Control groups.

Parameter	TMR group (n = 35)		Δ	p	Control group (n = 39)		Δ	p	Hedges’ g	
	Pre, median [IQR] (range)	Post, median [IQR] (range)			Pre, median [IQR] (range)	Post, median [IQR] (range)			TMR	Ctrl
SDNN, ms*	23 [17–27.5] (11–78)	30 [24–38.5] (13–64)	+7	<0.001	31 [22–38.5] (8–60)	25 [17.5–29.5] (6–55)	−6	<0.001	0.70	0.85
RMSSD, ms*	12 [9.5–16] (4–35)	19 [14–22.5] (3–55)	+7	<0.001	18 [14–25] (2–54)	13 [8–16.5] (2–49)	−5	<0.001	0.79	0.85
SI, units*	422 [345–818] (40–1439)	253 [179–391] (75–723)	−169	<0.001	257 [178.5–507] (60–1860)	431 [297–890.5] (62–1732)	+174	<0.001	0.86	0.73
HR, bpm*	76 [70–84] (57–100)	69 [65–77] (55–105)	−7	0.019	74 [67–81] (51–112)	80 [72.5–85.5] (49–118)	+6	<0.001	0.41	0.65
pNN20, %	5 [2–18] (0–58)	22 [11–38.5] (0–60)	+17	<0.001	18 [9.5–32] (0–74)	10 [2–14.5] (0–72)	−8	<0.001	0.90	0.80
SDSD, ms	7 [6–10] (2–20)	12 [8.5–14] (2–43)	+5	<0.001	11 [8.5–16.5] (2–31)	8 [5–11] (1–29)	−3	<0.001	0.63	0.70
Entropy†	0.28 [0.115–0.425] (0–0.7)	0.39 [0.275–0.575] (0.01–0.74)	+0.11	<0.001	0.39 [0.22–0.495] (0–0.83)	0.24 [0.07–0.37] (0–0.78)	−0.15	<0.001	0.73	0.80
IVR, units	661 [589.5–1141] (82–1812)	442 [281–707] (140–1274)	−219	<0.001	426 [292.5–759] (142–1965)	671 [543–1264] (152–1992)	+245	<0.001	0.77	0.69
HF, ms^2^	45.12 [20.98–100.7] (4.28–404.6)	117.8 [59.02–184.7] (0.76–625.8)	+72.66	<0.001	107.4 [49.64–175.4] (2.08–517.9)	47.87 [20.11–113.2] (1.77–441.2)	−59.51	<0.001	0.60	0.62
Op. Reg. Ctrl^‡^	58 [50.5–63.5] (43–78)	70 [60.5–74] (46–93)	+12	<0.001	67 [59.5–76] (37–86)	60 [51.5–65] (34–80)	−7	<0.001	1.16	1.08
Reg. Reserve^‡^	53 [38–62.5] (21–78)	67 [59–72.5] (23–82)	+14	<0.001	62 [51.5–70] (23–81)	55 [41–65.5] (22–76)	−7	0.001	0.91	0.60
Compl. Reg. Idx^‡^	58 [47–65] (35–79)	69 [62.5–75.5] (35–87)	+11	<0.001	67 [59.5–73.5] (29–84)	59 [47–67] (33–79)	−8	<0.001	1.12	0.88

Values are medians with interquartile range [IQR] and (minimum–maximum); recordings are 5-min supine. Within-group Pre–Post comparisons: Wilcoxon signed-rank test. Δ, change in median (Post − Pre). Effect size: Hedges’ g (paired). SDNN, standard deviation of NN, intervals; RMSSD, root mean square of successive differences; SI, baevsky stress index; HR, heart rate; pNN20, proportion of successive NN, differences >20 ms; SDSD, standard deviation of successive differences; IVR, index of vegetative regulation; HF, high-frequency power; HR, values in this table are derived from NN (normal-to-normal) intervals. † Entropy is the Harmony device approximate-entropy index.

^‡^ Op. Reg. Ctrl (operational regulatory control), Reg. Reserve (regulatory reserve) and Compl. Reg. Idx (complex regulatory index) are proprietary Harmony/OKR, indices (0–100), not standard international HRV, parameters. Pre-treatment values in this table are computed on the paired complete-case sample (TMR n, 35, Control n = 39) and therefore differ slightly from the baseline medians reported in Table 2, which are based on the full allocated cohort (TMR n, 37, Control n = 40).

* Primary outcomes. Cols 2–5: TMR, group; Cols 6–9: Control group. g = Hedges’ g (absolute). TMR, group: improvement (↑HRV, ↓SI); Control group: deterioration (↓HRV, ↑SI).

**TABLE 5:** ECG parameter changes in TMR and Control groups.

Parameter	TMR group (n = 35)		Δ	p	Control group (n = 39)		Δ	p	Hedges’ g	
	Pre [IQR](range)	Post [IQR](range)			Pre [IQR](range)	Post [IQR](range)			TMR	Ctrl
HR, bpm	77 [71–84] (57–100)	69 [65–77] (55–105)	−8	0.010	74 [67–81] (51–112)	80 [72.5–85.5] (49–118)	+6	<0.001	0.45	0.65
QT interval, s	0.392 [0.3755–0.409] (0.32–0.453)	0.401 [0.3865–0.419] (0.355–0.476)	+0.009	0.006	0.392 [0.374–0.4145] (0.345–0.461)	0.386 [0.3705–0.4005] (0.338–0.449)	−0.006	0.054	0.48	0.32
QTc (Bazett), s	0.437 [0.421–0.4535] (0.398–0.493)	0.436 [0.4235–0.447] (0.39–0.496)	−0.001	0.925	0.439 [0.428–0.4465] (0.399–0.489)	0.444 [0.4275–0.462] (0.391–0.513)	+0.005	0.005	0.05	0.50
QTcF (Fridericia), s	0.422 [0.4107–0.4357] (0.37–0.465)	0.427 [0.415–0.4317] (0.383–0.482)	+0.005	0.309	0.425 [0.413–0.432] (0.38–0.466)	0.422 [0.414–0.4325] (0.383–0.481)	−0.003	0.483	0.16	0.13
Tp–Te interval, s	0.084 [0.0785–0.092] (0.07–0.148)	0.085 [0.082–0.0935] (0.06–0.156)	+0.001	0.120	0.082 [0.078–0.092] (0.068–0.212)	0.08 [0.076–0.087] (0.058–0.212)	−0.002	0.514	0.08	0.02
QRS duration, s	0.099 [0.092–0.104] (0.062–0.12)	0.105 [0.096–0.11] (0.058–0.12)	+0.006	0.148	0.102 [0.094–0.106] (0.066–0.13)	0.1 [0.095–0.108] (0.066–0.114)	−0.002	0.965	0.32	0.11
PQ interval, s	0.156 [0.142–0.176] (0.11–0.19)	0.156 [0.14–0.1675] (0.116–0.188)	+0	0.112	0.16 [0.152–0.1825] (0.11–0.23)	0.168 [0.154–0.181] (0.118–0.212)	+0.008	0.827	0.16	0.10
Macruz index	1.75 [1.455–2.14] (0.37–2.81)	1.75 [1.515–2.095] (−17.79–4.27)	+0	0.159	1.75 [1.23–2.32] (0.8–33)	1.84 [1.28–2.6] (0.68–49.5)	+0.09	0.384	0.10	0.16
Myocardial state index	62 [54–65] (44–86)	63 [55–67] (47–79)	+1	0.231	63 [57–70] (39–81)	62 [56–72] (42–80)	−1	0.891	0.20	0.03
K1 = (PQ+QTc)/RR	0.7435 [0.6823–0.8595] (0.578–0.996)	0.69 [0.6318–0.78] (0.541–1.09)	−0.0535	0.013	0.731 [0.6495–0.8115] (0.5–0.987)	0.776 [0.725–0.885] (0.455–0.995)	+0.045	<0.001	0.39	0.65
Golden ratio index	0.875 [0.5725–0.9775] (0.25–1.53)	0.955 [0.755–1.105] (0.16–1.61)	+0.08	0.030	0.87 [0.655–1.1] (0.09–1.71)	0.62 [0.485–0.9] (0.06–2.05)	−0.25	<0.001	0.38	0.62
T-symm. by deriv. (aVL)	0.59 [0.195–0.87] (0–1.67)	0.6 [0.13–0.67] (0–0.98)	+0.01	0.014	0.62 [0.25–0.66] (0–0.89)	0.62 [0.495–0.73] (0–0.95)	+0	0.198	0.37	0.21
T-wave amplitude, aVF, µV	128 [62–190] (-65–355)	122 [83–204] (-44–306)	−6	0.268	146 [82–202] (-18–379)	136 [88–180] (0–377)	−10	0.858	0.02	0.28
QRS–T angle, frontal, °	14 [8–42] (0–103)	14 [7–39] (2–116)	+0	0.868	18 [10–33] (1–120)	18 [12–28] (1–134)	+0	0.950	0.12	0.10

Within-group pre–post ECG, changes; 5-min supine recordings. Values are medians with interquartile range [IQR] and (minimum–maximum). Within-group comparisons: Wilcoxon signed-rank test. Δ, change in median (Post − Pre); effect size: Hedges’ g (paired). HR, heart rate; QTc, rate-corrected QT (Bazett); QTcF, rate-corrected QT (Fridericia); Tp–Te, peak-to-end of T wave; K1, composite conduction–repolarization index. HR, values in this table are derived from ECG QRS, detection. The myocardial state index and Golden ratio index are proprietary Harmony device indices, not standard ECG, parameters; values reported for exploratory purposes.

Parameters with p < 0.05 in within-TMR OR, within-Control comparison (paired Wilcoxon signed-rank test). Δ = Post − Pre median. Hedges’ g computed on paired differences (small-sample bias-corrected). * denotes p < 0.05. One TMR, baseline recording with an internally inconsistent derived-parameter export (QTcF, Tp–Te, QRS, duration, HF-signs index, and the Golden ratio index all implausible despite a normal QT/QTc, for the same beat) was excluded from these five derived indices only; the participant’s HR, QT, QTc, and all other parameters were retained. Excluding this record left the reported medians and IQR, materially unchanged and narrowed the reported ranges accordingly. The Macruz index [P/(PQ−P)] is a ratio that becomes numerically unstable when the P-wave duration approaches the PQ, interval; the occasional extreme or negative values (e.g., the negative minimum and large maxima shown in the range) reflect this mathematical instability of the index rather than a physiological measurement and should be interpreted with caution.

#### 3.3.1 TMR group

Significant improvements were observed in all four primary HRV outcomes—SDNN, RMSSD, Stress Index, and heart rate. SDNN increased by 30% (23 → 30 ms; p < 0.001; g = 0.70), RMSSD by 58% (12 → 19 ms; p < 0.001; g = 0.79), SI decreased by 40% (422 → 253; p < 0.001; g = 0.86, large effect), and the HRV-derived heart rate fell from 76 to 69 bpm (p = 0.019; g = 0.41), consistent with the parallel ECG-derived heart-rate reduction described below. Among secondary outcomes, approximate entropy (ApEn) increased by 39% (0.28 → 0.39; p < 0.001). This shift indicates greater irregularity of the cardiac interval series; however, an isolated increase in a single entropy measure reflects greater complexity of cardiac dynamics rather than necessarily greater complexity of autonomic regulation, and in signals dominated by stochastic fluctuation may index randomness rather than physiological organization. It should therefore not be equated with enhanced regulatory capacity in the absence of complementary nonlinear analyses. Composite regulatory indices improved with large effect sizes (Operational Regulatory Control: +12 points, g = 1.16; Regulatory Reserve: +14, g = 0.91). HF power increased substantially (+72.7 ms^2^; p < 0.001), consistent with increased vagally mediated spectral modulation.

The ECG-derived heart rate decreased significantly from baseline (77 → 69 bpm; Δ = −8 bpm; p = 0.010; Hedges’ g = 0.45), consistent with the concurrent RMSSD increase and with increased vagally mediated modulation of sinoatrial rhythm. The absolute QT interval also increased significantly (0.392 → 0.401 s; p = 0.006; g = 0.48), a physiologically expected consequence of the HR reduction—longer diastolic intervals accommodate a longer ventricular repolarization sequence. Critically, neither QTc (0.437 → 0.436 s; p = 0.925) nor QTcF (0.422 → 0.427 s; p = 0.309) changed significantly, confirming that rate-corrected repolarization duration was stable. The cardiac cycle proportionality index K1 decreased significantly (0.744 → 0.690; p = 0.013; g = 0.39), indicating a change in this software-derived, exploratory index; this finding may partly reflect the concurrent heart-rate reduction. The software-derived golden ratio index increased significantly (0.88 → 0.96; p = 0.030; g = 0.38). T-wave symmetry in lead aVL changed significantly (0.59 → 0.60; p = 0.014; g = 0.38), representing a small change in an exploratory repolarization morphology index. Other ECG morphological parameters (QRS duration, PQ interval, Tp–Te, Macruz index) did not change significantly, indicating stable conduction properties throughout the intervention.

#### 3.3.2 Control group

During the same observation period, participants receiving standard care without TMR showed worsening of several HRV indices. SDNN decreased by 19% (31 → 25 ms; p < 0.001; g = 0.85), RMSSD by 28% (18 → 13 ms; p < 0.001; g = 0.85), SI increased by 68% (257 → 431; p < 0.001), and entropy decreased (0.39 → 0.24; p < 0.001; g = 0.80, moderate effect). Baevsky Functional State classification worsened (median 2 → 3; p = 0.005). This pattern may reflect persistent autonomic burden, treatment heterogeneity, or unmeasured contextual stressors.

In contrast to the TMR group, the control group showed a significant increase in ECG-derived HR (74 → 80 bpm; p < 0.001; g = 0.65), consistent with the parallel worsening of sympathovagal balance indices (SDNN, RMSSD, SI). The Bazett-corrected QTc increased significantly (0.439 → 0.444 s; p = 0.005; g = 0.50), whereas the Fridericia-corrected QTcF did not change significantly (0.425 → 0.422 s; p = 0.483) — a pattern consistent with overcorrection by the Bazett formula under the concurrent heart-rate increase, rather than a genuine change in rate-corrected repolarization duration. The frontal-plane QRS–T angle did not change significantly within groups (TMR 14 → 14°, p = 0.87; Control 18 → 18°, p = 0.95), and T-wave amplitude in lead aVF likewise showed no significant within-group shift; between-group differences in frontal-plane T-vector orientation were numerical and non-significant, indicating no robust progressive shifts in ventricular repolarization geometry. Other morphological parameters remained stable.

Rate-corrected HRV analysis. Because resting HR is a physiological confounder of HRV rather than an outcome in itself (Sacha, 2014), we repeated the within- and between-group comparisons using RR-normalized indices (RMSSD/RR and SDNN/RR, RR = 60000/HR) in the complete valid analysis sample (35 TMR, 39 Control). Within the TMR group, the increase in RMSSD/RR remained significant after rate correction (median 0.0137 → 0.0222; Wilcoxon p < 0.001), consistent in direction with the significant increase in raw RMSSD (p < 0.001; Table 4), and the SDNN/RR increase likewise remained significant (0.0276 → 0.0357; p < 0.001), in line with the raw SDNN result (p < 0.001; Table 4). Within the Control group, both raw and rate-corrected RMSSD and SDNN decreased significantly (raw SDNN p < 0.001, raw RMSSD p < 0.001; Table 4), indicating that the control-group deterioration is also not simply a rate-dependent artifact. At the between-group, post-treatment comparison, rate correction attenuated but did not abolish the difference: raw RMSSD (TMR vs. Control, p = 0.001; Table 6) remained significant after correction for RMSSD/RR (p = 0.007), and raw SDNN (p = 0.008; Table 6) remained nominally significant for SDNN/RR (p = 0.047), alongside a significant between-group difference in post-treatment HR itself (HRV-derived, 69 vs. 80 bpm; p = 0.001; Table 6). Taken together, these findings indicate that the reported within-group and between-group HRV differences are not primarily artifacts of concurrent heart-rate change, although part of the magnitude of the between-group post-treatment difference is attributable to the concurrent HR difference and the rate-corrected effect sizes should be regarded as the more conservative estimate of the autonomic effect. Because ratio-based HRV normalization (RMSSD/RR, SDNN/RR) and covariate-based heart-rate adjustment (rank-based ANCOVA; see Table 7) answer related but not identical statistical questions, the two sensitivity analyses were interpreted jointly rather than as competing conclusions. The RMSSD/RR and SDNN/RR analyses indicate that the direction and statistical signal of the between-group HRV difference persist after simple rate normalization. Table 7 presents the rank-based ANCOVA sensitivity analysis on the complete-case subsample used in the original analytical pipeline (TMR n = 33, Control n = 34); this ANCOVA has not been recomputed on the updated n = 35 TMR/39 Control analytical sample used elsewhere in this revision, and its descriptive medians therefore do not exactly match Tables 4, 6. Subject to that caveat, the ANCOVA indicated that a larger share of the between-group post-treatment difference in SDNN, RMSSD, HF, and ApEn is statistically explained by the concurrent heart-rate covariate. Taken together, these complementary analyses support an autonomic component that is not entirely explained by concurrent heart-rate differences, while indicating that the between-group magnitude should be interpreted conservatively, and that the Baevsky Stress Index difference is the most heart-rate-independent of the primary outcomes.

**TABLE 6:** Post-treatment intergroup comparison: TMR vs. Control: HRV parameters.

Parameter	Control post (n = 39)	TMR post (n = 35)	Δ	p	Hedges’ g	Category
	Median [IQR] (range)	Median [IQR] (range)				
SDNN, ms*	25 [17.5–29.5] (6–55)	30 [24–38.5] (13–64)	+5	0.008	0.63	Moderate
RMSSD, ms*	13 [8–16.5] (2–49)	19 [14–22.5] (3–55)	+6	0.001	0.62	Moderate
SI, units*	431 [297–890.5] (62–1732)	253 [179–391] (75–723)	−178	<0.001	0.86	Large
HR, bpm*	80 [72.5–85.5] (49–118)	69 [65–77] (55–105)	−11	0.001	0.69	Moderate
pNN20, %	10 [2–14.5] (0–72)	22 [11–38.5] (0–60)	+12	<0.001	0.83	Large
Entropy^†^	0.24 [0.07–0.37] (0–0.78)	0.39 [0.275–0.575] (0.01–0.74)	+0.15	0.001	0.80	Moderate
IVR, units	671 [543–1264] (152–1992)	442 [281–707] (140–1274)	−229	0.003	0.80	Moderate
Op. Reg. Ctrl^‡^	60 [51.5–65] (34–80)	70 [60.5–74] (46–93)	+10	0.001	0.84	Large
Compl. Reg. Idx^‡^	59 [47–67] (33–79)	69 [62.5–75.5] (35–87)	+10	<0.001	0.82	Large
Reg. Reserve^‡^	55 [41–65.5] (22–76)	67 [59–72.5] (23–82)	+12	0.002	0.74	Moderate
Baevsky FS	3 [2–5] (0–7)	2 [1–2] (0–7)	−1	0.002	0.72	Moderate

Post-treatment between-group comparison; 5-min supine recordings. Values are medians with interquartile range [IQR] and (minimum–maximum). Between-group comparison: Mann–Whitney U-test. Effect size: Hedges’ g (unpaired); category per |g| thresholds (0.2 small, 0.5 moderate, 0.8 large). Abbreviations as in Table 2; Baevsky FS, Baevsky functional state score.

^‡^ Op. Reg. Ctrl (operational regulatory control), Compl. Reg. Idx (complex regulatory index) and Reg. Reserve (regulatory reserve) are proprietary Harmony/OKR, indices (0–100), not standard international HRV, parameters.

^†^ approximate-entropy index; descriptive values are identical to those in Table 4. Table 7 presents a heart-rate-adjusted sensitivity analysis on a smaller complete-case subsample (TMR n, 33, Control n = 34) and its unadjusted p-values and descriptive medians do not exactly match this table.

* Primary outcomes. Δ = TMR − Control. Positive Δ for HRV; negative Δ for SI/IVR/HR, indicate better outcomes in TMR.

**TABLE 7:** Exploratory heart-rate-adjusted sensitivity analysis: post-treatment between-group HRV comparison in the original complete-case subsample (TMR n = 33, Control n = 34; a smaller subsample than the TMR n = 35, Control n = 39 sample used elsewhere in this revision), unadjusted and adjusted for heart rate (rank-based ANCOVA on the 5-min supine recordings).

Parameter	TMR post, median [IQR]	Control post, median [IQR]	p (unadj.)	p (HR-adj.)
SDNN, ms	30 [25–39]	24 [15–30]	0.0074	0.185
RMSSD, ms	18.5 [14–24]	13 [8–17]	0.0042	0.148
Stress index	267.5 [178–387]	437 [294–830]	0.0007	0.024
ApEn	0.395 [0.25–0.57]	0.23 [0.08–0.37]	0.0055	0.227
HF, ms^2^	112.07 [57.5–190.4]	46.69 [18.6–116.8]	0.0077	0.152
LF/HF	2.5 [1.8–3.3]	3.1 [1.7–3.8]	0.328	0.615
HR, bpm	69 [66–78]	79 [71–86]	0.0075	Covariate

After heart-rate adjustment, the between-group differences in SDNN, RMSSD, HF, and approximate entropy (ApEn) were attenuated to non-significance, whereas the Baevsky Stress Index remained significant (p = 0.024) — indicating that most of the between-group HRV advantage is heart-rate-mediated while the reduction in regulatory rigidity is at least partly heart-rate-independent. Values are medians with interquartile ranges (IQR). ApEn, approximate entropy; HF, high-frequency power; LF/HF, low-/high-frequency ratio. p (unadj.): Mann–Whitney U-test; p (HR-adj.): rank-based ANCOVA with heart rate as covariate. Descriptive medians and interquartile ranges are those of the original post-treatment complete-case subsample (TMR n = 33, Control n = 34) and do not exactly match the updated Tables 4, 6 (TMR n = 35, Control n = 39); this table has not been recomputed on the updated sample pending re-implementation of the rank-based ANCOVA.

### 3.4 Post-treatment intergroup comparison

Post-treatment differences between groups were significant across all primary and most secondary outcomes (Table 6), reflecting both improvement in TMR and deterioration in Control.

Notably, several HRV parameters in the post-treatment TMR group approached civilian reference values (SDNN 30 vs. 39 ms; RMSSD 19 vs. 24 ms), while the Control group diverged further from civilian norms. HF power increased substantially in the TMR group (45.1 → 117.8 ms^2^; p < 0.001) and decreased in Control (107.4 → 47.9 ms^2^; p < 0.001). LF/HF ratio did not change significantly in either group (p > 0.1), suggesting absolute power shifts rather than spectral balance redistribution. DFA exponent showed a slight non-significant decrease in TMR (−0.05; p = 0.19), consistent with a shift toward more flexible regulation.

Post-treatment intergroup differences were detected in several ECG parameters (Table 8). The most prominent finding was the between-group difference in HR (TMR: 69 bpm vs. Control: 80 bpm; p = 0.001; g = 0.69), consistent with the concurrent HRV differences and consistent with greater vagal modulation in the TMR group. The QT interval was also significantly longer in the TMR group (0.401 vs. 0.386 s; p = 0.008; g = 0.62); because the rate-corrected QTcF did not differ between groups (p = 0.665), this QT difference is attributable to the heart-rate difference rather than to intrinsic repolarization change. Tp–Te was modestly longer in the TMR group (0.086 vs. 0.080 s; p = 0.015; g = 0.15), but the effect size was negligible and the finding should be interpreted cautiously, particularly given the absence of QTcF prolongation or arrhythmic events. In terms of repolarization morphology, the TMR group showed numerically lower ST-segment deviation at J+80 ms in lead I (0.030 vs. 0.045 mV; p = 0.147, non-significant; g = 0.39), a numerically lower integral ST–T index in lead aVL (50.0 vs. 69.5; p = 0.190, non-significant; g = 0.31), numerically lower T-wave amplitude in lead I (167 vs. 197 μV; p = 0.24, non-significant; g = 0.19), and no significant difference in T-wave symmetry in lead aVL (0.60 vs. 0.62; p = 0.41, non-significant; g = 0.20). None of these lead-specific morphological indices reached significance on the consistent post-treatment sample, in contrast to the significant cardiac-cycle proportionality indices (K1, golden ratio) reported below. The cardiac cycle proportionality index K1 was lower in the TMR group (0.688 vs. 0.776; p = 0.002; g = 0.64), and the golden ratio index was higher (0.96 vs. 0.62; p = 0.003; g = 0.62, moderate effect), indicating between-group differences in exploratory software-derived cardiac-cycle proportionality indices. The PQ interval was shorter in the TMR group (0.156 vs. 0.168 s; p = 0.016; g = 0.57, moderate); both group medians remained well within the normal range of atrioventricular conduction, and the physiological significance of this difference is uncertain in the absence of a baseline between-group difference in PQ. Notably, composite myocardial state index, HF signs index, QRS duration, and Macruz index did not differ significantly between groups at follow-up, suggesting that structural and conduction properties of the myocardium were similarly preserved in both groups.

**TABLE 8:** Post-treatment intergroup comparison: TMR vs. Control: ECG parameters.

Parameter	Control post (n = 39)	TMR post (n = 35)	Δ	p	Hedges’ g	Category
	Median [IQR] (range)	Median [IQR] (range)				
HR, bpm	80 [72.5–85.5] (49–118)	69 [65–77] (55–105)	−11	0.001	0.69	Moderate
QT interval, s	0.386 [0.3705–0.4005] (0.338–0.449)	0.401 [0.3865–0.419] (0.355–0.476)	+0.015	0.008	0.62	Moderate
QTc (Bazett), s	0.444 [0.4275–0.462] (0.391–0.513)	0.436 [0.4235–0.447] (0.39–0.496)	−0.008	0.144	0.30	Small
QTcF (Fridericia), s	0.422 [0.414–0.4325] (0.383–0.481)	0.427 [0.415–0.433] (0.383–0.482)	+0.005	0.665	0.05	Negligible
Tp–Te interval, s	0.08 [0.076–0.087] (0.058–0.212)	0.086 [0.082–0.094] (0.06–0.156)	+0.006	0.015	0.15	Negligible
QRS duration, s	0.1 [0.095–0.108] (0.066–0.114)	0.106 [0.096–0.111] (0.058–0.12)	+0.006	0.218	0.29	Small
PQ interval, s	0.168 [0.154–0.181] (0.118–0.212)	0.156 [0.14–0.1675] (0.116–0.188)	−0.012	0.016	0.57	Moderate
Macruz index	1.84 [1.28–2.6] (0.68–49.5)	1.75 [1.515–2.095] (−17.79–4.27)	−0.09	0.935	0.48	Small
Myocardial state index	62 [56–72] (42–80)	63 [55–67] (47–79)	+1	0.598	0.13	Negligible
ST dev. J+80 ms (lead I), mV	0.045 [0.021–0.072] (−0.030–0.118)	0.030 [0.016–0.061] (−0.017–0.095)	−0.015	0.147	0.39	Small
Integral ST–T index (aVL)	69.5 [44.0–85.8] (10.0–100.0)	50.0 [10.0–83.0] (10.0–100.0)	−19.5	0.190	0.31	Small
K1 = (PQ+QTc)/RR	0.776 [0.725–0.885] (0.455–0.995)	0.688 [0.63–0.776] (0.541–1.09)	−0.088	0.002	0.64	Moderate
Golden ratio index	0.62 [0.485–0.9] (0.06–2.05)	0.96 [0.76–1.13] (0.16–1.61)	+0.34	0.003	0.62	Moderate
T-symm. by deriv. (aVL)	0.62 [0.5025–0.73] (0–0.95)	0.6 [0.13–0.67] (0–0.98)	−0.02	0.403	0.22	Small
T-wave amplitude, lead I, µV	197 [144–268] (61–337)	167 [121–245] (46–431)	−30	0.243	0.19	Negligible
T-wave amplitude, lead III, µV	40 [-44–107] (-125–294)	47 [-46–128] (-169–224)	+7	0.965	0.14	Negligible
T-wave amplitude, aVL, µV	88 [36–136] (-72–225)	79 [0–116] (-52–285)	−9	0.353	0.10	Negligible
Alpha-T angle, frontal, °	38 [18–55] (-4–74)	41 [17–65] (-11–75)	+3	0.539	0.11	Negligible

Post-treatment between-group ECG, comparison; 5-min supine recordings. Values are medians with interquartile range [IQR] and (minimum–maximum). Between-group comparison: Mann–Whitney U-test; effect size: Hedges’ g (unpaired); category per |g| thresholds. HR, heart rate; QTc, rate-corrected QT (Bazett); QTcF, rate-corrected QT (Fridericia); Tp–Te, peak-to-end of T wave; ST, dev., ST-segment deviation; ISTT, integral ST–T, shape index; K1, composite conduction–repolarization index. The myocardial state index and Golden ratio index are proprietary Harmony device indices, not standard ECG, parameters; values reported for exploratory purposes.

* p < 0.05 (Mann–Whitney U test). Δ = TMR, Post − Control Post. Effect-size categories: small (≥0.20), moderate (≥0.50), large (≥0.80), very large (≥1.20). ns = not significant. The Macruz index [P/(PQ−P)] becomes numerically unstable when the P-wave duration approaches the PQ, interval; the extreme values shown in the range reflect this mathematical instability rather than a physiological measurement, which also explains the discordance between its non-significant rank-based p-value and its parametric effect size. It should be interpreted with caution. The Tp–Te interval likewise contains extreme high-end values (range up to 0.212 s against a median of approximately 0.08 s); because the rank-based Mann–Whitney test is sensitive to a distributional shift whereas Hedges’ g is computed on means and is attenuated by such outliers, a significant p-value may coincide with a negligible effect size for this parameter. The Tp–Te difference should therefore be regarded as statistically detectable but of negligible magnitude.

#### 3.4.1 HRV–ECG coupling

**FIGURE 2:**
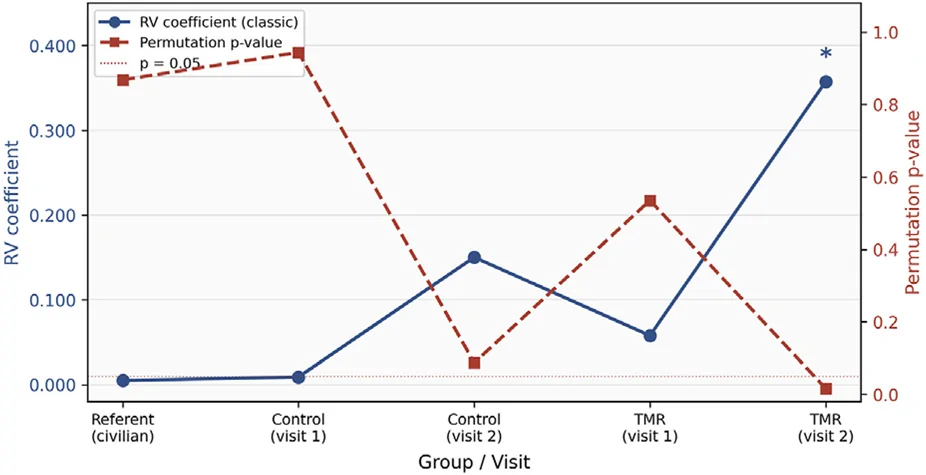
Multivariate HRV–ECG association (RV coefficient) by group and measurement time point. Left axis (blue, circles): classic RV coefficient; right axis (red, squares, dashed): permutation p-value. Asterisk (*): p < 0.05 (permutation test, 5,000 permutations). Note: the classic RV is subject to upward small-sample bias in high-dimensional settings; bias-corrected estimates require the cleaned dataset parameters. Control: standard care group; TMR: Technique of Mental Relaxation group.

To further characterize the relationship between autonomic and electrophysiological adaptation, a multivariate RV-coefficient analysis was performed to quantify the global association between the HRV and ECG parameter blocks across groups and time points (RV coefficient; Escoufier, 1973; Robert and Escoufier, 1976; significance assessed by permutation testing; Josse et al., 2008; small-sample bias correction following Smilde et al., 2009; Josse and Holmes, 2016) (Figure 2). In the civilian reference group and in both military groups at baseline, the RV coefficient was negligible and non-significant (RV ≤ 0.009; all permutation p > 0.53), indicating near-absent inter-block coupling. After the treatment period, the control group showed a numerically higher but non-significant coefficient (RV = 0.150; p = 0.087). In contrast, the TMR group at the post-treatment visit demonstrated a statistically significant inter-block association (RV = 0.357; permutation p = 0.016), indicating that HRV and ECG parameter profiles co-varied more strongly than expected by chance. The bias-corrected modified RV coefficient (Smilde et al., 2009) closely matched the classic estimate (RV2 = 0.360 vs. RV = 0.357), and the permutation null expectation was low (E0 = 0.076), yielding a bias-adjusted value of RV − E0 = 0.282; the bootstrap 95% confidence interval for the modified RV excluded zero [0.062–0.636]. Statistical significance therefore persisted after correction for small-sample bias (permutation p = 0.011). After partialling out heart rate, the block-level coefficient was no longer significant (partial RV = 0.062; p = 0.48), indicating that the global coupling was predominantly heart-rate-mediated, consistent with the parameter-level analysis below. The predominantly rate-dependent nature of this coupling is discussed below (Complementary ECG Findings and HRV–ECG Coupling).

To examine further whether post-intervention changes in autonomic regulation were accompanied by correlated changes in ECG morphological markers, pairwise Spearman correlations with FDR correction were computed between the HRV and ST-T parameter blocks for each group separately (625 pairs predefined per group, of which 500 were testable per group after excluding one ST-T sub-parameter absent from the source records; Table 9).

**TABLE 9:** Significant HRV–ECG morphological associations in the TMR group at post-treatment visit (FDR q < 0.05; n = 35).

ECG parameter (lead)	HRV parameter	ρ	FDR q	Partial ρ*	p (partial)
Integral ST-T form (II)	pNN50, %	+0.59	0.028	+0.51	0.0016
Integral ST-T form (II)	SDSD, ms	+0.60	0.028	+0.53	0.0010
T-wave symmetry (max deriv., II)	pNN50, %	−0.57	0.028	−0.52	0.0013
ST displacement +0.08 s (II)	Entropy	+0.60	0.028	+0.45	0.0071
T-wave symmetry (max deriv., aVF)	pNN50, %	−0.57	0.028	−0.53	0.0010
ST displacement +0.08 s (aVF)	Entropy	+0.57	0.028	+0.43	0.0106
T-wave symmetry (max deriv., II)	TP, ms^2^	−0.56	0.030	—	—
ST displacement +0.08 s (II)	RMSSD, ms	+0.56	0.033	+0.41	0.0146
ST displacement +0.08 s (II)	pNN20, %	+0.53	0.037	—	—
ST displacement +0.08 s (II)	pNN50, %	+0.54	0.037	—	—
Integral ST-T form (II)	HF, ms^2^	+0.54	0.037	—	—
T-wave symmetry (max deriv., II)	SDSD, ms	−0.54	0.037	—	—
T-wave symmetry (max deriv., II)	HF, ms^2^	−0.53	0.037	—	—
ST displacement +0.08 s (aVF)	RMSSD, ms	+0.53	0.037	+0.39	0.0210
Integral ST-T form (II)	TP, ms^2^	+0.53	0.037	—	—
Integral ST-T form (II)	RMSSD, ms	+0.53	0.037	—	—
ST displacement +0.08 s (aVF)	pNN50, %	+0.52	0.039	—	—
ST displacement +0.08 s (II)	SDSD, ms	+0.52	0.042	—	—
ST displacement +0.08 s (II)	HF, ms^2^	+0.51	0.050	—	—

* Partial ρ: Spearman correlation after residualising both variables on HR., Reported for the three most prominent ECG, parameters only; HRV, parameters abbreviations; SDNN, standard deviation of NN, intervals; RMSSD, root mean square of successive RR, differences; SI, Baevsky stress index; pNN50 — proportion of successive RR, differences >50 ms; pNN20 — > 20 ms; SDSD, standard deviation of successive RR, differences; HF, high-frequency spectral power (0.15–0.40 Hz); LF, low-frequency spectral power (0.04–0.15 Hz); TP, total spectral power; HR, heart rate. No significant HRV–ST-T, morphological associations were detected in the control group or civilian reference group (all FDR q > 0.05).

In the civilian reference group (n = 89) and in the military control group at the post-treatment visit (n = 39), no statistically significant associations between HRV indices and ST-T morphological parameters were detected after FDR correction (q > 0.05 for all 500 pairs tested in the control group; the civilian reference group was not re-analyzed in this revision). In contrast, 19 significant HRV–ECG morphological pairs (FDR q < 0.05) were identified exclusively in the TMR group at the post-treatment visit (n = 35). One of the five predefined ST-T sub-parameter types (a categorical T-wave-height indicator), represented as one column in each of the five recorded leads, was not populated in the source records and could not be tested; this removed five of the 25 ST-T columns, leaving 25 HRV indices × 20 ST-T parameters = 500 of the 625 predefined pairs available for FDR correction per group. All significant pairs were confined to leads II (15 pairs) and aVF (4 pairs), corresponding to the inferior and inferior-lateral myocardial territory.

Three ECG parameters drove the significant associations: the ST segment displacement at 0.08 s after the J-point (leads II and aVF), the integral index of ST-T morphology (lead II), and T-wave symmetry assessed by the ratio of maximal derivatives (leads II and aVF). The strongest associations were observed between the integral ST-T form index (lead II) and SDSD (ρ = +0.60, FDR q = 0.028) and pNN50 (ρ = +0.59, FDR q = 0.028), and between ST displacement (lead II) and Entropy (ρ = +0.60, FDR q = 0.028) and RMSSD (ρ = +0.56, FDR q = 0.033). Inverse associations were found between T-wave symmetry (lead II) and pNN50 (ρ = −0.57, FDR q = 0.028) and total spectral power TP (ρ = −0.56, FDR q = 0.030), with a parallel inverse association in lead aVF (T-wave symmetry vs. pNN50: ρ = −0.57, FDR q = 0.028).

To evaluate the independence of these associations from heart rate, partial Spearman correlations were computed after residualising both variables on HR, for the three ECG parameter types identified above. All eight examined associations were attenuated but remained nominally significant after HR adjustment: integral ST-T form–pNN50 (partial ρ = +0.51, p = 0.002), integral ST-T form–SDSD (partial ρ = +0.53, p = 0.001), ST displacement–Entropy (lead II, partial ρ = +0.45, p = 0.007; lead aVF, partial ρ = +0.43, p = 0.011), ST displacement–RMSSD (lead II, partial ρ = +0.41, p = 0.015; lead aVF, partial ρ = +0.39, p = 0.021), and T-wave symmetry–pNN50 (lead II, partial ρ = −0.52, p = 0.001; lead aVF, partial ρ = −0.53, p = 0.001). T-wave symmetry associations were not attenuated more than the ST-segment or integral-form associations, so the associations should be regarded collectively as partially HR-independent rather than as evidence of a specific dissociation between rate-dependent and rate-independent ECG markers. Summary data are presented in Table 9; full pairwise results are available from the corresponding author upon request.

### 3.5 Blood pressure

Median arterial BP was within normal limits at both time points and did not change significantly in either group (p > 0.1). This reflects group medians recorded on ongoing antihypertensive treatment where indicated and should not be read as absence of hypertension in the cohort: a single office measurement ≥140/90 mmHg was documented in 52% of the TMR group and 62% of the control group (Supplementary Table S1), and physician-coded arterial hypertension was present in 24% and 39%, respectively (Table 1). Baseline systolic BP was 126 ± 8 mmHg in TMR and 125 ± 10 mmHg in Control. A non-significant trend toward slight systolic BP reduction (3–5 mmHg) was observed in both groups, consistent with evidence that longer-duration breathing training (>4–8 weeks) is typically required for clinically meaningful antihypertensive effects.

### 3.6 Heart-rate-adjusted between-group comparison

To address the potential confounding influence of resting heart rate on HRV metrics (Sacha and Pluta, 2008; Sacha, 2014), the post-treatment between-group comparison (TMR vs. Control) was repeated with heart rate entered as a covariate using rank-based analysis of covariance (Table 7). After heart-rate adjustment, the between-group differences in SDNN (p = 0.185), RMSSD (p = 0.148), HF power (p = 0.152), and approximate entropy (ApEn; p = 0.227) were attenuated to non-significance, whereas the difference in the Baevsky Stress Index remained significant (p = 0.024). This dissociation indicates that a substantial portion of the apparent between-group HRV advantage in the TMR group is mediated by the concurrent lower heart rate, while the reduction in regulatory rigidity captured by the Stress Index is at least partly independent of heart rate.

## 4 Discussion

This study examined HRV and ECG changes associated with adding a structured breathing–meditation protocol, referred to as TMR, to standard rehabilitation in combat-exposed military personnel. The findings suggest a more favorable autonomic trajectory in the adjunctive intervention group, reflected by increases in HRV parameters and reductions in stress-related indices compared with the standard-treatment-only group.

### 4.1 Baseline autonomic dysregulation

Both military groups demonstrated profoundly impaired autonomic regulation compared with civilians, with marked differences across several HRV parameters. SDNN values of 23–29 ms—approximately 59%–74% of civilian values (39 ms) — indicate statistically significant between-group reductions in overall autonomic adaptability; because validated clinical cut-off values for SDNN, RMSSD, SI, and IVR are not established for this population, these differences are reported as statistically (not clinically) significant. RMSSD values of 12–16 ms, approximately 50%–67% of civilian norms (24 ms), are consistent with reduced vagally mediated cardiac modulation. The SI values (347–422 vs. 187) and IVR (589–664 vs. 298) are consistent with a more sympathetically predominant or less flexible autonomic pattern. These findings are consistent with previous studies of HRV depression in PTSD and chronic stress populations (Chalmers et al., 2014; Hauschildt et al., 2011; Matsyshyn et al., 2023).

The entropy reduction (0.22–0.29 vs. 0.45 in civilians) is particularly noteworthy, suggesting a qualitative shift toward rigid, less adaptive autonomic dynamics. This is consistent with the “complexity loss” hypothesis (Goldberger et al., 2002), wherein chronic allostatic load reduces the capacity of regulatory systems to respond flexibly to environmental demands.

### 4.2 Mechanisms of improvement in the TMR group

The observed improvements in the TMR group may be explained by several well-characterized physiological mechanisms. Slow-paced breathing at six cycles/min (0.1 Hz) is thought to engage the baroreflex resonance mechanism: cyclical intrathoracic pressure oscillations synchronize with the intrinsic ∼10-s period of the arterial baroreflex arc, amplifying both respiratory sinus arrhythmia and baroreflex gain (Lehrer and Gevirtz, 2014; Vaschillo et al., 2006). This may result in enhanced vagal efferent outflow to the sinoatrial node, manifesting as increased RMSSD and HF power.

Diaphragmatic breathing may further contribute by stimulating vagal afferent pathways through pulmonary stretch receptor activation (Hering–Breuer reflex), which projects to the nucleus tractus solitarius (NTS) and subsequently to parasympathetic efferent nuclei.

The observational meditation component may contribute through central “top-down” mechanisms. Previous neurophysiological studies have suggested that meditative practices are associated with activation of prefrontal cortical networks and improved inhibitory control over limbic stress-response circuits (Thayer and Lane, 2009; Thayer et al., 2012). Although these central mechanisms were not directly measured in the present study, the observed improvements in HRV and entropy are consistent with enhanced autonomic flexibility and reduced physiological stress, as predicted by the neurovisceral integration model.

The increase in RMSSD (58%) and entropy (39%) may indicate not only higher HRV magnitude but also a shift toward greater autonomic complexity and regulatory flexibility, consistent with the “complexity loss” hypothesis (Goldberger et al., 2002); however, this interpretation remains indirect and should be confirmed in studies specifically designed to assess autonomic mechanistic pathways.

### 4.3 Deterioration in the control group

The worsening of autonomic parameters in the Control group during the observation period is noteworthy and requires cautious interpretation. Several factors may explain this finding: (1) combat-related autonomic dysregulation may follow a persistent or progressive trajectory without targeted autonomic retraining; (2) certain psychotropic medications, including antidepressants and sedative agents, have been associated with reduced HRV in some populations (Kemp et al., 2010; O’Regan et al., 2015); (3) environmental stressors within the rehabilitation setting, such as anticipation of return to duty or continued psychological burden, may contribute to persistent sympathetic activation; and (4) without specific autonomic retraining, the combat-induced hypervigilance and sympathetic “set point” shift may persist despite symptom-focused treatment. These findings may indicate that standard rehabilitation protocols do not consistently address autonomic dysregulation in this population and could potentially be complemented by targeted autonomic retraining interventions.

### 4.4 Complementary ECG findings and HRV–ECG coupling

ECG analysis revealed complementary findings. At baseline, the TMR group—which had worse HRV—also showed more pronounced ECG repolarization differences from the civilian reference, including reduced T-wave amplitudes and altered T-vector orientation, consistent with sympathetically driven repolarization changes; the Tp–Te interval, by contrast, did not differ from the civilian reference at baseline (0.082 s in all three groups; Table 3). Following TMR, the principal ECG effect was HR reduction accompanied by a significant increase in the absolute QT interval; critically, rate-corrected intervals (QTc, QTcF) remained stable, supporting the absence of clinically relevant QTc prolongation in the intervention group. In the Control group, the Bazett-corrected QTc increased modestly but significantly (0.439 → 0.444 s; p = 0.005; g = 0.50) in the context of a heart-rate increase, whereas the Fridericia-corrected QTcF did not change significantly—a pattern consistent with overcorrection by the Bazett formula under the concurrent heart-rate increase rather than a genuine change in rate-corrected repolarization duration, and this finding should therefore be interpreted cautiously rather than as evidence of clinically relevant repolarization deterioration. At the post-treatment visit, 17 of 39 control patients (44%) exceeded the clinical QTc threshold of 450 ms (6 of 39, 15%, exceeded 470 ms; maximum 0.513 s), compared with seven of 35 (20%) in the TMR group (3 of 35, 9%, exceeded 470 ms; maximum 0.496 s), suggesting a potentially unfavorable repolarization trajectory in the control group that warrants confirmation in a randomized study. Post-treatment between-group divergence was confined to the cardiac-cycle proportionality indices (K1, p = 0.002; golden ratio index, p = 0.003) and the PQ interval (p = 0.016), whereas the lead-specific repolarization morphology indices did not differ significantly between groups—integral ST–T shape index in lead aVL (p = 0.076) and T-wave symmetry in lead aVL (p = 0.403) (Table 8). Because K1 and the golden ratio index contain the RR interval by construction, these differences are largely rate-carrying rather than independent markers of repolarization morphology. To our knowledge, few controlled studies have examined ECG-derived repolarization indices in the context of breathing–meditation rehabilitation among combat-exposed military personnel, and the present findings may contribute to this underexplored area.

The post-intervention increase in multivariate HRV–ECG association in the TMR group is consistent with several physiologically plausible mechanisms. Slow diaphragmatic breathing at near-resonant frequencies (∼0.1 Hz) synchronizes autonomic oscillations through the baroreflex loop, creating correlated fluctuations across cardiovascular parameters simultaneously captured in both the HRV and ECG domains (Lehrer and Gevirtz, 2014; Zaccaro et al., 2018). Increased vagally mediated cardiac modulation, suggested by higher RMSSD and HF power, may influence sinus-node dynamics and rate-sensitive ECG indices (Sacha, 2014). Additionally, a possible reduction in sympathetic predominance or regulatory rigidity may decrease inter-individual dispersion of repolarization heterogeneity, further increasing statistical coherence between the two parameter blocks.

However, the individual-pair analysis revealed that the strongest HRV–ECG correlations in the post-treatment TMR group involved exclusively rate-carrying ECG indices: the composite ratio K1 = (PQ + QTc)/RR, which contains the RR interval in its denominator by construction (Pearson r = 0.97 with HR), and the “Golden Ratio” index (r = −0.96 with HR, consistent across all groups). Genuine myocardial morphology markers—ST-segment shift, T-wave symmetry indices, and integral ST-T form parameters—did not reach the strong-coupling threshold in any group. This pattern suggests that the observed RV increase is substantially mediated by the TMR-induced HR reduction (ECG-derived, 77 → 69 bpm) rather than by novel structural autonomic–myocardial coupling, an interpretation consistent with the absence of between-group differences in rate-corrected repolarization parameters (Table 8). Whether a genuine coupling between autonomic regulation and myocardial electrophysiology exists beyond the rate effect warrants future investigation using HR-adjusted multivariate methods.

The exclusive emergence of significant HRV–ST-T morphological coupling in the post-treatment TMR group warrants mechanistic consideration. All significant pairs involved vagal or vagally-associated HRV indices—RMSSD, pNN50, SDSD, HF power, total spectral power, and Entropy—which primarily reflect beat-to-beat parasympathetic modulation of the sinoatrial node (Task Force, 1996). The anatomical substrate for this association is established: parasympathetic fibres innervate not only the sinoatrial and atrioventricular nodes but also the ventricular myocardium, where acetylcholine modulates action potential duration and the morphology of myocardial repolarization (Billman, 2013). Higher RMSSD and HF power are consistent with increased vagally mediated heart-rate modulation in the TMR group, and can therefore directly influence ST-T morphology through acetylcholine-mediated shortening of ventricular action potential duration and reduction of repolarization heterogeneity across the myocardial wall (Antzelevitch and Burashnikov, 2011). The positive direction of the ST displacement association—higher RMSSD and pNN50 correlating with a more positive J+0.08 s ST level—is consistent with the well-described pattern of enhanced parasympathetic tone producing mild ST elevation in inferior leads, an electrophysiological signature of vagally mediated early repolarization (Noseworthy et al., 2011; Tikkanen et al., 2009).

Critically, the leading integral ST-T form, ST-segment displacement, and T-wave symmetry associations with vagal HRV indices all partially survived HR adjustment (partial ρ = 0.39–0.53, p = 0.001–0.021), indicating that this HRV–ECG coupling is not fully attributable to the concurrent heart-rate reduction, without a clear dissociation between individual ECG morphology markers. T-wave symmetry and duration are known to be strongly rate-dependent (QT/RR relationship; Sacha, 2014), so some HR-mediated contribution to all reported associations, including T-wave symmetry, cannot be excluded; the present partial-correlation analysis indicates only that a residual HR-independent component is statistically detectable, not which specific electrophysiological mechanism underlies it. The concentration of significant associations in leads II and aVF—territories reflecting the inferior left ventricular wall—may reflect the anatomical distribution of parasympathetic ventricular innervation, which has been shown to predominate in inferior regions of the left ventricle in humans (Randall et al., 1996). Preganglionic parasympathetic cardiac neurons originate predominantly in the nucleus ambiguus, with a smaller contribution from the dorsal motor nucleus of the vagus (Geis and Wurster, 1980). Taken together, these findings suggest that the TMR protocol, by being associated with increased vagally mediated modulation, may be associated with a modest, partially HR-independent influence on inferior-wall myocardial repolarization markers, though the present exploratory, hypothesis-generating analysis cannot establish a specific causal mechanism. The absence of any significant HRV–ST-T associations in the control group—despite a comparable sample size (n = 39) and the same ECG recording conditions—further supports the specificity of this finding to the breathing-meditation intervention.

Interpreted within the framework of Network Physiology, these findings position HRV–ECG coupling as a measure of interaction between two physiological sub-systems—the autonomic control network and the cardiac electrophysiological sub-system—rather than as a property of either in isolation (Bashan et al., 2012; Bartsch et al., 2015; Ivanov, 2021). The post-intervention emergence of significant coupling in the TMR group, absent in the control and civilian reference groups, is consistent with the central premise of Network Physiology that physiological state is encoded not only in the output of individual systems but also in the strength and topology of their dynamic interactions. That most of this coupling was heart-rate-mediated, while a smaller vagally driven ST–T component partially persisted after heart-rate adjustment, illustrates the multiscale and multi-mechanistic nature of such inter-system links and underscores the value of network-based analyses that examine several physiological domains jointly rather than in parallel. These results should be regarded as exploratory and hypothesis-generating, and they motivate future studies applying time-resolved network-physiological methods to autonomic–cardiac coupling during breathing–meditation interventions.

### 4.5 Comparison with published evidence

The meta-analysis by Fincham et al. (2023) reported moderate effect sizes (g = 0.32–0.40) for breathing interventions on stress and anxiety in predominantly civilian populations. The larger effect sizes observed in the present study (g = 0.62–0.86 for primary HRV outcomes) may reflect the greater baseline autonomic impairment in combat-exposed individuals, allowing for greater physiological improvement. Shyam et al. (2022) reported SDNN increases following a 4-week resonance breathing protocol in healthy young adults; the present findings extend this to a clinically affected population within a shorter timeframe. The work of Gerbarg et al. (2019) on the Breath–Body–Mind (BBM) technique for veterans with PTSD is the closest published parallel to the present study: like TMR, BBM combines slow diaphragmatic breathing (∼5–6 breaths/min) with a meditative attentional component and targets autonomic rebalancing in trauma-exposed populations. Both approaches report parasympathetic enhancement and symptom reduction. However, the present study differs in three respects that strengthen its inferential value: it uses a concurrent control group rather than a single-arm design, it applies objective HRV/ECG endpoints rather than predominantly self-report outcomes, and it is conducted in an actively combat-exposed military cohort. Direct quantitative comparison remains limited by differences in breathing cadence prescription, session structure, and outcome batteries.

### 4.6 Clinical implications

The findings suggest potential benefits of integrating structured breathing–meditation protocols into military rehabilitation programs. Key advantages include: a structured, reproducible protocol teachable in a single session; no requirement for specialized equipment; no adverse events were reported during the observation period; possibility of objective monitoring using HRV parameters; and potential for sustained self-application beyond clinical settings. HRV monitoring may serve as an objective tool for tracking autonomic changes during rehabilitation and may help inform individualized rehabilitation strategies.

### 4.7 Strengths and limitations

#### 4.7.1 Strengths

The study provides objective HRV and ECG assessment in a real-world clinical setting, evaluates a combat-exposed military population that remains underrepresented in controlled autonomic regulation studies, includes a civilian reference group for normative benchmarking, employs a comprehensive panel of time-domain, spectral, nonlinear, and composite HRV parameters, uses standardized effect size reporting, and provides detailed clinical characterization of participants including comorbidities and concurrent pharmacotherapy. The study also provides exploratory ECG analysis in a context where controlled evidence is very limited; subtle ECG changes may complement HRV findings and merit further investigation in future studies.

#### 4.7.2 Limitations

Several limitations should be considered. First, the study employed a controlled but non-randomized design, with group allocation based on clinical feasibility, which introduces potential selection bias. The TMR group had worse baseline HRV than the Control group, which may partially explain the larger magnitude of improvement through regression to the mean, although the parallel deterioration in the Control group argues against this as the sole explanation. Regression to the mean cannot be fully excluded; the findings should therefore be interpreted as preliminary and hypothesis-generating, and future randomized studies with baseline-matched groups or ANCOVA-adjusted primary analyses are needed to confirm the observed between-group differences. Additionally, the ECG analyses were performed on the same 5-min supine recordings as the HRV analyses (post-treatment: TMR n = 35, Control n = 39; baseline: TMR n = 37, Control n = 40); isolated per-parameter missingness reflects occasional technical exclusions due to ECG recording quality; per-parameter complete-case counts are reported in Supplementary Table S3. Second, the intervention period was relatively short (14 days), and long-term sustainability of the observed effects remains unclear. Third, concurrent pharmacotherapy may have influenced HRV parameters; available chart review did not indicate an obvious medication-class imbalance, but complete person-level medication data were unavailable for formal group-by-group comparison, and residual medication-related confounding cannot be excluded. Fourth, the sample consisted exclusively of male participants, limiting generalizability to female military personnel. Fifth, the study focused primarily on physiological autonomic outcomes and did not include a parallel standardized symptom outcome analysis (e.g., DASS-21, PCL-5) as a co-primary endpoint; available psychological screening data (NSI, PCL-5) were not systematically repeated at discharge for all participants. Sixth, composite regulatory indices, while clinically informative, require further independent validation. Seventh, smoking status, office blood pressure, and body mass index were retrieved from the structured clinical registry and did not differ significantly between the groups (Supplementary Table S1); however, because these variables were incompletely recorded (for example, smoking status was documented for 29 of 37 TMR-group and 29 of 40 control participants), they were not entered as covariates in the HRV models, and residual confounding by anthropometric and lifestyle factors cannot be fully excluded. Eighth, although slow breathing was central to the intervention, formal cardiovascular autonomic reflex testing—including the expiratory:inspiratory (E:I) ratio and heart-rate response to paced deep breathing—was not performed, so the diagnostic autonomic response to the manoeuvre was not quantified. Ninth, the interval from the primary traumatic event to hospitalization varied widely (1–120 months) and prior hospitalizations were common; disease chronicity and severity were not entered as covariates in the HRV models and may act as residual confounders. Finally, the inclusion age criterion was broadened during recruitment from an initial 35–60-year target to 23–61 years to reflect actual clinical eligibility; this is now stated explicitly in the inclusion criteria rather than treated as a *post hoc* discrepancy.

Future studies should employ randomized controlled designs with appropriate blinding (e.g., sham breathing control), longer follow-up periods, larger sample sizes, inclusion of female participants, integration of subjective psychological measures alongside HRV analysis, and investigation of long-term functional outcomes.

## 5 Conclusion

This study addressed whether adding a structured breathing–meditation protocol (TMR) to standard rehabilitation is associated with changes in HRV and ECG-derived indices of autonomic regulation in combat-exposed military personnel. Both military groups entered the study with pronounced autonomic dysregulation relative to a civilian reference sample, confirming that combat exposure in this cohort is associated with a substantial burden of impaired autonomic and cardiac-electrophysiological regulation. Over the 14-day observation period, the group receiving TMR as an adjunct to standard care showed a more favorable autonomic trajectory across HRV time-domain, spectral, and composite regulatory indices, accompanied by a parallel decrease in resting heart rate on both the HRV-derived and ECG-derived measurements, whereas the standard-care-only group showed the opposite pattern. Exploratory ECG analysis in the TMR group showed parallel, rate-related changes in selected repolarization and cardiac-cycle proportionality indices, with no evidence of clinically relevant QTc prolongation.

Taken together, the sensitivity analyses indicate that this between-group difference is not an artifact of concurrent heart-rate change. Exploratory ECG analysis showed no evidence of clinically relevant repolarization prolongation in the intervention group, and the emergence of HRV–ECG coupling specifically in the TMR group—partly, though not fully, explained by heart rate—suggests that the intervention may act at the level of dynamic integration between the autonomic and cardiac sub-systems, consistent with a Network Physiology perspective, rather than on isolated organ outputs alone.

These findings best regarded as preliminary, hypothesis-generating evidence rather than definitive proof of efficacy.

If confirmed in adequately powered randomized controlled trials with longer follow-up, blinding, and inclusion of female participants, a structured breathing–meditation protocol such as TMR could offer a low-cost, equipment-free, and readily teachable adjunct to standard rehabilitation for combat-exposed military personnel, with HRV serving as an objective, non-invasive tool for monitoring autonomic recovery. Beyond its immediate rehabilitative value, sustained normalization of autonomic and HRV–ECG coupling parameters may carry broader implications for long-term cardiovascular risk and stress resilience in this population, a question that future studies with extended follow-up should directly address.

## Data Availability

The datasets generated and/or analyzed during the current study are not publicly available due to privacy and military-related confidentiality considerations but may be made available from the corresponding author upon reasonable request and subject to ethical approval.
