# Supplementary material for: Heart rate variability, ECG minor changes, and HRV–ECG coupling associated with an adjunctive structured breathing–meditation protocol in combat-exposed military personnel
Source: Front Netw Physiol. 2026 Aug 13;6:1912231. doi: 10.3389/fnetp.2026.1912231 (PMC13518136; doi:10.3389/fnetp.2026.1912231)
Supplement: Supplementary file 1 [file Table1.docx]

**Supplementary Materials**

*Heart Rate Variability, ECG Minor Changes, and HRV–ECG Coupling Associated With an Adjunctive Structured Breathing–Meditation Protocol in Combat-Exposed Military Personnel*

**Supplementary Methods S1. Signal Processing**

**Artifact detection and correction**

Each RR interval was flagged as an artifact when it deviated from the local mean of the surrounding intervals by more than 20%. Flagged intervals were replaced using cubic interpolation from adjacent valid intervals. The percentage of corrected or excluded intervals was recorded for every recording. Recordings with more than 5% edited intervals were excluded from spectral analysis.

**Resampling**

The non-uniform RR-interval series was converted into an evenly sampled time series through cubic spline interpolation, followed by resampling at 4 Hz. This resampling rate preserves the high-frequency component of the spectrum up to 0.4 Hz without introducing significant distortion.

**Spectral estimation**

Spectral estimation was performed using the Fast Fourier Transform. Welch's method was applied, averaging the periodogram across overlapping segments to reduce estimator variance. Each segment consisted of 256 points, corresponding to approximately 64 seconds at a 4 Hz sampling rate. A Hanning window was applied to each segment to minimize spectral leakage at the segment boundaries. Consecutive segments overlapped by 50%.

**Detrending and stationarity**

Before spectral estimation, the linear trend was removed from each RR-interval time series by subtracting a first-order polynomial fit. This detrending step eliminated slow drift unrelated to autonomic modulation and satisfied the stationarity assumption required for FFT-based analysis. Formal stationarity testing (e.g., trend exclusion, run-length or variance-based tests) beyond visual tachogram inspection was not performed and is noted as a limitation.

**Frequency bands**

Spectral power was integrated over the conventional frequency bands: very-low-frequency (VLF, 0.0033–0.04 Hz), low-frequency (LF, 0.04–0.15 Hz), and high-frequency (HF, 0.15–0.40 Hz), following Task Force (1996) standards; the LF/HF ratio was computed from the LF and HF absolute powers.

**R-peak detection**

R-peak detection is performed within the proprietary firmware of the "Oracul/Harmony" ECG-HRV analysis system and is not independently configurable by the end user; the vendor-specific detection algorithm is not publicly documented.

**Supplementary Table S1. Baseline lifestyle, blood pressure, and body-mass characteristics by group.**

| **Characteristic** | **TMR group (n = 37)** | **Control group (n = 40)** | **p** |
| --- | --- | --- | --- |
| Current smoker,ᵃ n (%) | 25/29 (86%) | 20/29 (69%) | 0.207 (Fisher) |
| Elevated office blood pressure (≥140/90 mmHg),ᵇ n (%) | 14/27 (52%) | 21/34 (62%) | 0.603 (Fisher) |
| BMI, kg/m², median [IQR]ᶜ | 25.3 [22.9–29.4] | 26.3 [24.3–28.8] | 0.499 (MWU) |

*ᵃ Current smoker includes cigarette and electronic-cigarette/vaping use; entries left blank were treated as unknown and excluded from the denominator. Recording coverage: 29/37 (TMR) and 29/40 (Control).*

*ᵇ Defined by a single office blood-pressure measurement ≥ 140/90 mmHg; a structured physician-coded hypertension diagnosis was not available in the registry for all participants. Recording coverage: 27/37 (TMR) and 34/40 (Control).*

*ᶜ Computed from recorded height and weight. Coverage: 37/37 (TMR) and 39/40 (Control). Values are medians with interquartile range [IQR]; between-group comparison, Mann–Whitney U-test (MWU).*

**Supplementary Table S2. Concurrent pharmacotherapy in the military cohort.**

| **Medication class** | **Agent(s)** | **Typical dose** | **Proportion of cohort** |
| --- | --- | --- | --- |
| Atypical antipsychotic / anxiolytic | Quetiapine (Quetiron) | 25–200 mg at bedtime | >70% |
| GABAergic anticonvulsant | Pregabalin | 75–150 mg/day | ≈55% |
| Melatonin-based hypnotic | Melatonin (Vita-Melatonin) | standard dose | >50% |
| Antidepressant (SSRI) | Paroxetine, escitalopram, sertraline, fluvoxamine | standard dose | ≈40% |
| Mood stabilizer | Valproate | standard dose | ≈20% |
| Antidepressant (trazodone) | Trazodone | standard dose | ≈15% |
| Neuroprotective agents | Citicoline, B-vitamin complex (vitaxon), betahistine | standard dose | not quantified |
| Antidepressant (SNRI) | Venlafaxine, duloxetine | standard dose | isolated cases |
| Antihypertensive | per documented hypertension | individualized | not quantified |

*Note. Percentages reflect the proportion of the combined military cohort (TMR + Control) receiving each medication class, as documented in clinical records. Medication classes were similarly distributed between the TMR and Control groups, with no significant intergroup differences in the proportions of patients receiving antidepressants, antipsychotics, or anticonvulsants. Per-patient, dose-level medication data were not fully digitized in the structured study database, so an independently verifiable group-by-group (TMR vs. Control) numerical breakdown with formal statistical comparison could not be produced from the database and is not presented; the cohort-level proportions above are as reported in the clinical chart review underlying the main text.*

**Supplementary Table S3. Complete-case sample sizes for ECG-derived parameters.**

| **ECG parameter** | **TMR pre (of 37)** | **TMR post (of 35)** | **Control pre (of 40)** | **Control post (of 39)** | **Civilian (of 89)** |
| --- | --- | --- | --- | --- | --- |
| HR, bpm | 36 | 35 | 39 | 39 | 89 |
| QT interval, s | 36 | 35 | 39 | 39 | 89 |
| QTc (Bazett), s | 36 | 35 | 39 | 39 | 89 |
| QTcF (Fridericia), s | 35 | 35 | 39 | 39 | 89 |
| Tp–Te interval, s | 35 | 35 | 39 | 39 | 89 |
| QRS duration, s | 35 | 35 | 39 | 39 | 89 |
| PQ interval, s | 36 | 35 | 39 | 39 | 89 |
| Macruz index | 36 | 35 | 39 | 39 | 89 |
| Myocardial state index | 35 | 35 | 39 | 39 | 89 |
| Integral ST–T index, lead II | 36 | 35 | 39 | 39 | 89 |
| Integral ST–T index, lead III | 36 | 35 | 39 | 39 | 89 |
| Integral ST–T index, aVL | 36 | 35 | 39 | 39 | 89 |
| ST deviation J+80 ms, lead I | 35 | 35 | 39 | 39 | 89 |
| T-wave amplitude, lead I | 36 | 35 | 39 | 39 | 89 |
| T-wave amplitude, lead III | 36 | 35 | 39 | 39 | 89 |
| T-wave amplitude, aVL | 36 | 35 | 39 | 39 | 89 |
| T-wave amplitude, aVF | 36 | 35 | 39 | 39 | 89 |
| Alpha-T angle, frontal | 36 | 35 | 39 | 39 | 89 |
| QRS–T angle, frontal | 36 | 35 | 39 | 39 | 89 |
| HF signs index | 35 | 35 | 39 | 39 | 89 |
| K1 = (PQ+QTc)/RR | 34 | 35 | 37 | 35 | 89 |
| Golden ratio index | 35 | 35 | 39 | 39 | 89 |
| T-symmetry, aVL | 35 | 35 | 38 | 36 | 89 |

*Note. Values are the number of participants with a non-missing value for each parameter, out of the group total shown in the column header. The post-treatment analytical sample used throughout this revision is TMR n = 35, Control n = 39; the pre-treatment (baseline) columns are shown against the officially allocated cohort (TMR n = 37, Control n = 40), though one participant per group lacks any five-minute supine baseline recording in the source records and is therefore absent from every pre-treatment row (achievable baseline maximum: TMR 36, Control 39). Missingness reflects isolated technical exclusions (e.g., transient signal-quality issues affecting a specific derived index) rather than systematic non-recording; most parameters have no missing values. One TMR baseline recording with an internally inconsistent derived-parameter export was excluded from five indices only (QTcF, Tp–Te, QRS duration, HF signs index, and the Golden ratio index); the participant's other parameters, including the post-treatment visit, were retained.*
