# Supplementary material for: Heart rate variability, ECG minor changes, and HRV–ECG coupling associated with an adjunctive structured breathing–meditation protocol in combat-exposed military personnel
Source: Front Netw Physiol. 2026 Aug 13;6:1912231. doi: 10.3389/fnetp.2026.1912231 (PMC13518136; doi:10.3389/fnetp.2026.1912231)
Supplement: Supplementary file 2 [file Table2.docx]

***Table 1.*** *Group-specific prevalence of principal comorbidities and additional baseline characteristics, based on structured chart review (n = 66 with complete medical records). p-values are two-sided Fisher's exact test (categorical rows). None reached significance at ‎α‎ = 0.05, consistent with broadly comparable baseline comorbidity burden between groups, although the table was not powered for formal between-group inference****.***

| **Characteristic** | **TMR (n = 33)** | **Control (n = 33)** | **p (Fisher)** |
| --- | --- | --- | --- |
| Arterial hypertension (any grade) | 8 (24%) | 13 (39%) | 0.290 |
| Hypertension grade II–III (severe) | 3 (9%) | 7 (21%) | 0.303 |
| Hypercholesterolemia (documented) | 5 (15%) | 8 (24%) | 0.537 |
| Degenerative spine disease | 10 (30%) | 10 (30%) | 1.000 |
| Any documented prior hospitalization | 23 (70%) | 24 (73%) | 1.000 |
| Age, years (mean ± SD) | 47.6 ± 5.9 | 45.7 ± 7.7 | — |
| Time from trauma to hospitalization, months, median [range]ᵃ | 13 [1–120] (n=19) | 19 [1–96] (n=16) | — |

*ᵃ Time-to-hospitalization interval was explicitly documented in the medical record for a subset of participants (19/33 TMR, 16/33 Control); values are not available for the remainder and are not imputed. Body mass index, smoking status, and office blood pressure were available from the clinical registry for most participants and are reported by group in Supplementary Table S1; they were not incorporated into this table. Specific echocardiographic diagnoses (hypertensive heart disease, myocardial fibrosis, mitral/tricuspid valve insufficiency, diastolic dysfunction) were documented in narrative clinical records but were not consistently coded as structured, group-attributable variables across all charts; they are therefore described qualitatively at the cohort level only and are not broken out by group in this table.*

***Table 2.*** *Baseline HRV parameters in military groups vs. civilian reference (medians).*

| **Parameter** | **Control (n = 40)** | **TMR (n = 37)** | **Civilian (n = 89)** | **Δ Ctrl vs Civ** | **p** | **Δ TMR vs Civ** | **p** |
| --- | --- | --- | --- | --- | --- | --- | --- |
|  | **median [IQR] (range)** | **median [IQR] (range)** | **median [IQR] (range)** |  |  |  |  |
| SDNN, ms* | 29 [22–38] (8–60) | 23 [17–28] (11–78) | 39 [31–47] (15–91) | 10 | <0.0001 | 16 | <0.0001 |
| RMSSD, ms* | 16 [14–25] (2–54) | 12 [9–16] (4–77) | 24 [17–31] (8–109) | 8 | <0.0001 | 12 | <0.0001 |
| SI, units* | 347 [179–500] (60–1860) | 422 [361–823] (40–1439) | 187 [125–301] (26–771) | −160 | <0.0001 | −235 | <0.0001 |
| HR, bpm* | 76.5 [66.0–81.0] (51.0–112.0) | 78 [71.0–85.0] (57.0–100.0) | 73 [67.0–79.0] (56.0–105.0) | −3.5 | 0.057 | −5 | 0.013 |
| pNN20, % | 12.5 [10–32] (0–74) | 6 [2–17] (0–81) | 33 [19–50] (2–83) | 20.5 | <0.0001 | 27 | <0.0001 |
| SDSD, ms | 10 [9–16] (2–31) | 7 [6–10] (2–46) | 15 [11–19] (5–85) | 5 | 0.0003 | 8 | <0.0001 |
| Entropy† | 0.29 [0.23–0.49] (0.00–0.83) | 0.22 [0.09–0.42] (0.00–0.86) | 0.45 [0.29–0.61] (0.02–0.85) | 0.16 | <0.0001 | 0.23 | <0.0001 |
| IVR, units | 588.5 [293–756] (142–1965) | 664 [584–1129] (82–1812) | 298 [214–488] (53–1037) | −290.5 | <0.0001 | −366 | <0.0001 |

*Baseline (pre-treatment) values; five-minute supine recordings (civilian reference: single measurement). Values are medians with interquartile range [IQR] and (minimum–maximum). Between-group comparisons vs. civilians: Mann–Whitney U-test. Δ, difference in medians. SDNN, standard deviation of NN intervals; RMSSD, root mean square of successive differences; SI, Baevsky stress index; HR, heart rate; pNN20, proportion of successive NN differences > 20 ms; SDSD, standard deviation of successive differences; IVR, index of vegetative regulation. † approximate-entropy index (Harmony device). Baseline medians in this table are computed on the full allocated cohort (Control n = 40, TMR n = 37); the corresponding baseline medians in Tables 4 and 6 are computed on the paired complete-case sample (Control n = 39, TMR n = 35) and therefore differ slightly, whereas interquartile ranges and ranges are essentially unchanged.*

** Primary outcomes. Δ = civilian median − military-group median.*

***Table 3****. Baseline ECG parameters in military groups vs. civilian reference*

| **Parameter** | **Control (n = 40)**  **median [IQR] (range)** | **TMR (n = 37)**  **median [IQR] (range)** | **Civilian (n = 89)**  **median [IQR] (range)** | **Δ Ctrl vs Civ** | **p** | **Δ TMR vs Civ** | **p** |
| --- | --- | --- | --- | --- | --- | --- | --- |
| HR, bpm | 73.0 [66.0–81.0] (51.0–112.0) | 77.0 [71.0–85.0] (57.0–100.0) | 73.0 [67.0–79.0] (56.0–105.0) | +0 | 0.783 | +4 | 0.103 |
| QT interval, s | 0.393 [0.374–0.416] (0.345–0.478) | 0.392 [0.376–0.405] (0.320–0.453) | 0.384 [0.367–0.400] (0.325–0.467) | +0.009 | 0.078 | +0.008 | 0.136 |
| QTc (Bazett), s | 0.440 [0.428–0.448] (0.399–0.501) | 0.443 [0.425–0.459] (0.398–0.493) | 0.427 [0.416–0.439] (0.366–0.479) | +0.013 | 0.001 | +0.016 | 0.002 |
| QTcF (Fridericia), s | 0.425 [0.413–0.433] (0.380–0.493) | 0.424 [0.411–0.436] (0.370–0.465) | 0.412 [0.400–0.421] (0.357–0.475) | +0.013 | <0.001 | +0.012 | 0.002 |
| Tp–Te interval, s | 0.082 [0.078–0.092] (0.068–0.212) | 0.082 [0.077–0.092] (0.066–0.148) | 0.082 [0.078–0.086] (0.068–0.106) | +0 | 0.428 | +0 | 0.316 |
| QRS duration, s | 0.102 [0.094–0.106] (0.066–0.130) | 0.098 [0.092–0.106] (0.062–0.120) | 0.102 [0.094–0.108] (0.080–0.128) | +0 | 0.980 | -0.002 | 0.371 |
| PQ interval, s | 0.160 [0.151–0.182] (0.110–0.230) | 0.156 [0.142–0.174] (0.110–0.190) | 0.152 [0.140–0.166] (0.108–0.220) | +0.008 | 0.009 | +0.004 | 0.290 |
| Macruz index | 1.77 [1.23–2.36] (0.80–33.00) | 1.75 [1.46–2.16] (0.37–7.70) | 1.37 [1.02–2.00] (0.54–28.00) | +0.4 | 0.008 | +0.38 | 0.003 |
| Myocardial state index | 63 [57–70] (39–81) | 62 [54–65] (44–86) | 64 [59–68] (43–77) | -1 | 0.990 | -2 | 0.133 |
| Integral ST–T index (lead II) | 68.5 [51.2–77.2] (12.0–93.0) | 66.0 [45.0–74.0] (0.1–96.0) | 62.0 [44.0–74.0] (10.0–100.0) | +6.5 | 0.305 | +4 | 0.824 |
| Integral ST–T index (lead III) | 23.5 [10.0–51.2] (10.0–75.0) | 22.0 [10.0–40.0] (0.0–100.0) | 11.0 [10.0–26.0] (10.0–93.0) | +12.5 | 0.005 | +11 | 0.012 |
| T-wave amplitude, lead I, µV | 204 [140–280] (36–374) | 155 [104–231] (0–467) | 309 [221–387] (68–709) | -105 | <0.001 | -154 | <0.001 |
| T-wave amplitude, lead III, µV | 45 [-44–113] (-176–296) | 53 [-28–122] (-185–265) | -32 [-137–82] (-341–255) | +77 | 0.006 | +85 | 0.010 |
| Alpha-T angle, frontal, ° | 40 [18–54] (-3–83) | 44 [20–57] (-39–68) | 22 [7–41] (-40–76) | +18 | 0.004 | +22 | 0.003 |
| QRS–T angle, frontal, ° | 18 [10–33] (1–120) | 14 [8–42] (0–103) | 26 [16–47] (0–102) | -8 | 0.084 | -12 | 0.059 |
| HF signs index (ECG) | 0.1 [0.1–0.2] (0.0–0.6) | 0.18 [0.11–0.24] (0.06–0.39) | 0.1 [0.1–0.2] (0.0–0.5) | +0 | 0.387 | +0.1 | 0.043 |

*Baseline (pre-treatment) ECG parameters; five-minute supine recordings (civilian reference: single measurement). Values are medians with interquartile range [IQR] and (minimum–maximum). Between-group comparisons vs. civilians: Mann–Whitney U-test. HR, heart rate; QTc, rate-corrected QT (Bazett); QTcF, rate-corrected QT (Fridericia); Tp–Te, peak-to-end of T wave; ST dev., ST-segment deviation; ISTT, integral ST–T shape index; K1, composite conduction–repolarization index. HR values in this table are derived from ECG QRS detection. The myocardial state index and Golden ratio index are proprietary Harmony device indices, not standard ECG parameters; values reported for exploratory purposes. Group headers indicate the allocated baseline cohorts (TMR n = 37, Control n = 40); parameter-specific available counts are reported in Supplementary Table S3, with a maximum of 36 TMR and 39 Control baseline ECG recordings.*

*Parameters with p < 0.05 in either military group vs. civilian comparison (Mann–Whitney U test, * denotes p < 0.05). Δ = group median − Civilian median. QTc/QTcF civilian reference from age-matched normative data (Task Force, 1996). ns = not significant or civilian reference unavailable for this proprietary parameter. One TMR baseline recording with an internally inconsistent derived-parameter export (QTcF, Tp–Te, QRS duration, HF-signs index, and the Golden ratio index all implausible despite a normal QT/QTc for the same beat) was excluded from these five derived indices only; the participant's HR, QT, QTc, and all other parameters were retained. Excluding this record left the reported medians and IQR materially unchanged and narrowed the reported ranges accordingly.*

***Table 4.*** *HRV changes in TMR and Control groups.*

| **Parameter** | **TMR group (n = 35)** | |  |  | **Control group (n = 39)** | |  |  | **Hedges’ g** | |
| --- | --- | --- | --- | --- | --- | --- | --- | --- | --- | --- |
|  | **Pre, median [IQR] (range)** | **Post, median [IQR] (range)** | **Δ** | **p** | **Pre, median [IQR] (range)** | **Post, median [IQR] (range)** | **Δ** | **p** | **TMR** | **Ctrl** |
| SDNN, ms* | 23 [17–27.5] (11–78) | 30 [24–38.5] (13–64) | +7 | <0.001 | 31 [22–38.5] (8–60) | 25 [17.5–29.5] (6–55) | −6 | <0.001 | 0.70 | 0.85 |
| RMSSD, ms* | 12 [9.5–16] (4–35) | 19 [14–22.5] (3–55) | +7 | <0.001 | 18 [14–25] (2–54) | 13 [8–16.5] (2–49) | −5 | <0.001 | 0.79 | 0.85 |
| SI, units* | 422 [345–818] (40–1439) | 253 [179–391] (75–723) | −169 | <0.001 | 257 [178.5–507] (60–1860) | 431 [297–890.5] (62–1732) | +174 | <0.001 | 0.86 | 0.73 |
| HR, bpm* | 76 [70–84] (57–100) | 69 [65–77] (55–105) | −7 | 0.019 | 74 [67–81] (51–112) | 80 [72.5–85.5] (49–118) | +6 | <0.001 | 0.41 | 0.65 |
| pNN20, % | 5 [2–18] (0–58) | 22 [11–38.5] (0–60) | +17 | <0.001 | 18 [9.5–32] (0–74) | 10 [2–14.5] (0–72) | −8 | <0.001 | 0.90 | 0.80 |
| SDSD, ms | 7 [6–10] (2–20) | 12 [8.5–14] (2–43) | +5 | <0.001 | 11 [8.5–16.5] (2–31) | 8 [5–11] (1–29) | −3 | <0.001 | 0.63 | 0.70 |
| Entropy† | 0.28 [0.115–0.425] (0–0.7) | 0.39 [0.275–0.575] (0.01–0.74) | +0.11 | <0.001 | 0.39 [0.22–0.495] (0–0.83) | 0.24 [0.07–0.37] (0–0.78) | −0.15 | <0.001 | 0.73 | 0.80 |
| IVR, units | 661 [589.5–1141] (82–1812) | 442 [281–707] (140–1274) | −219 | <0.001 | 426 [292.5–759] (142–1965) | 671 [543–1264] (152–1992) | +245 | <0.001 | 0.77 | 0.69 |
| HF, ms² | 45.12 [20.98–100.7] (4.28–404.6) | 117.8 [59.02–184.7] (0.76–625.8) | +72.66 | <0.001 | 107.4 [49.64–175.4] (2.08–517.9) | 47.87 [20.11–113.2] (1.77–441.2) | −59.51 | <0.001 | 0.60 | 0.62 |
| Op. Reg. Ctrl‡ | 58 [50.5–63.5] (43–78) | 70 [60.5–74] (46–93) | +12 | <0.001 | 67 [59.5–76] (37–86) | 60 [51.5–65] (34–80) | −7 | <0.001 | 1.16 | 1.08 |
| Reg. Reserve‡ | 53 [38–62.5] (21–78) | 67 [59–72.5] (23–82) | +14 | <0.001 | 62 [51.5–70] (23–81) | 55 [41–65.5] (22–76) | −7 | 0.001 | 0.91 | 0.60 |
| Compl. Reg. Idx‡ | 58 [47–65] (35–79) | 69 [62.5–75.5] (35–87) | +11 | <0.001 | 67 [59.5–73.5] (29–84) | 59 [47–67] (33–79) | −8 | <0.001 | 1.12 | 0.88 |

*Values are medians with interquartile range [IQR] and (minimum–maximum); recordings are five-minute supine. Within-group Pre–Post comparisons: Wilcoxon signed-rank test. Δ, change in median (Post − Pre). Effect size: Hedges’ g (paired). SDNN, standard deviation of NN intervals; RMSSD, root mean square of successive differences; SI, Baevsky stress index; HR, heart rate; pNN20, proportion of successive NN differences > 20 ms; SDSD, standard deviation of successive differences; IVR, index of vegetative regulation; HF, high-frequency power. HR values in this table are derived from NN (normal-to-normal) intervals. † Entropy is the Harmony device approximate-entropy index. ‡ Op. Reg. Ctrl (operational regulatory control), Reg. Reserve (regulatory reserve) and Compl. Reg. Idx (complex regulatory index) are proprietary Harmony/OKR indices (0–100), not standard international HRV parameters. Pre-treatment values in this table are computed on the paired complete-case sample (TMR n = 35, Control n = 39) and therefore differ slightly from the baseline medians reported in Table 2, which are based on the full allocated cohort (TMR n = 37, Control n = 40).*

** Primary outcomes. Cols 2–5: TMR group; Cols 6–9: Control group. g = Hedges’ g (absolute). TMR group: improvement (↑HRV, ↓SI); Control group: deterioration (↓HRV, ↑SI).*

***Table 5.*** *ECG parameter changes in TMR and Control groups.*

| **Parameter** | **TMR group (n = 35)** | |  |  | **Control group (n = 39)** | |  |  | **Hedges’ g** | |
| --- | --- | --- | --- | --- | --- | --- | --- | --- | --- | --- |
|  | **Pre [IQR](range)** | **Post [IQR](range)** | **Δ** | **p** | **Pre [IQR](range)** | **Post [IQR](range)** | **Δ** | **p** | **TMR** | **Ctrl** |
| HR, bpm | 77 [71–84] (57–100) | 69 [65–77] (55–105) | −8 | 0.010 | 74 [67–81] (51–112) | 80 [72.5–85.5] (49–118) | +6 | <0.001 | 0.45 | 0.65 |
| QT interval, s | 0.392 [0.3755–0.409] (0.32–0.453) | 0.401 [0.3865–0.419] (0.355–0.476) | +0.009 | 0.006 | 0.392 [0.374–0.4145] (0.345–0.461) | 0.386 [0.3705–0.4005] (0.338–0.449) | −0.006 | 0.054 | 0.48 | 0.32 |
| QTc (Bazett), s | 0.437 [0.421–0.4535] (0.398–0.493) | 0.436 [0.4235–0.447] (0.39–0.496) | −0.001 | 0.925 | 0.439 [0.428–0.4465] (0.399–0.489) | 0.444 [0.4275–0.462] (0.391–0.513) | +0.005 | 0.005 | 0.05 | 0.50 |
| QTcF (Fridericia), s | 0.422 [0.4107–0.4357] (0.37–0.465) | 0.427 [0.415–0.4317] (0.383–0.482) | +0.005 | 0.309 | 0.425 [0.413–0.432] (0.38–0.466) | 0.422 [0.414–0.4325] (0.383–0.481) | −0.003 | 0.483 | 0.16 | 0.13 |
| Tp–Te interval, s | 0.084 [0.0785–0.092] (0.07–0.148) | 0.085 [0.082–0.0935] (0.06–0.156) | +0.001 | 0.120 | 0.082 [0.078–0.092] (0.068–0.212) | 0.08 [0.076–0.087] (0.058–0.212) | −0.002 | 0.514 | 0.08 | 0.02 |
| QRS duration, s | 0.099 [0.092–0.104] (0.062–0.12) | 0.105 [0.096–0.11] (0.058–0.12) | +0.006 | 0.148 | 0.102 [0.094–0.106] (0.066–0.13) | 0.1 [0.095–0.108] (0.066–0.114) | −0.002 | 0.965 | 0.32 | 0.11 |
| PQ interval, s | 0.156 [0.142–0.176] (0.11–0.19) | 0.156 [0.14–0.1675] (0.116–0.188) | +0 | 0.112 | 0.16 [0.152–0.1825] (0.11–0.23) | 0.168 [0.154–0.181] (0.118–0.212) | +0.008 | 0.827 | 0.16 | 0.10 |
| Macruz index | 1.75 [1.455–2.14] (0.37–2.81) | 1.75 [1.515–2.095] (-17.79–4.27) | +0 | 0.159 | 1.75 [1.23–2.32] (0.8–33) | 1.84 [1.28–2.6] (0.68–49.5) | +0.09 | 0.384 | 0.10 | 0.16 |
| Myocardial state index | 62 [54–65] (44–86) | 63 [55–67] (47–79) | +1 | 0.231 | 63 [57–70] (39–81) | 62 [56–72] (42–80) | -1 | 0.891 | 0.20 | 0.03 |
| K1 = (PQ+QTc)/RR | 0.7435 [0.6823–0.8595] (0.578–0.996) | 0.69 [0.6318–0.78] (0.541–1.09) | −0.0535 | 0.013 | 0.731 [0.6495–0.8115] (0.5–0.987) | 0.776 [0.725–0.885] (0.455–0.995) | +0.045 | <0.001 | 0.39 | 0.65 |
| Golden ratio index | 0.875 [0.5725–0.9775] (0.25–1.53) | 0.955 [0.755–1.105] (0.16–1.61) | +0.08 | 0.030 | 0.87 [0.655–1.1] (0.09–1.71) | 0.62 [0.485–0.9] (0.06–2.05) | −0.25 | <0.001 | 0.38 | 0.62 |
| T-symm. by deriv. (aVL) | 0.59 [0.195–0.87] (0–1.67) | 0.6 [0.13–0.67] (0–0.98) | +0.01 | 0.014 | 0.62 [0.25–0.66] (0–0.89) | 0.62 [0.495–0.73] (0–0.95) | +0 | 0.198 | 0.37 | 0.21 |
| T-wave amplitude, aVF, µV | 128 [62–190] (-65–355) | 122 [83–204] (-44–306) | -6 | 0.268 | 146 [82–202] (-18–379) | 136 [88–180] (0–377) | -10 | 0.858 | 0.02 | 0.28 |
| QRS–T angle, frontal, ° | 14 [8–42] (0–103) | 14 [7–39] (2–116) | +0 | 0.868 | 18 [10–33] (1–120) | 18 [12–28] (1–134) | +0 | 0.950 | 0.12 | 0.10 |

*Within-group pre–post ECG changes; five-minute supine recordings. Values are medians with interquartile range [IQR] and (minimum–maximum). Within-group comparisons: Wilcoxon signed-rank test. Δ, change in median (Post − Pre); effect size: Hedges’ g (paired). HR, heart rate; QTc, rate-corrected QT (Bazett); QTcF, rate-corrected QT (Fridericia); Tp–Te, peak-to-end of T wave; K1, composite conduction–repolarization index. HR values in this table are derived from ECG QRS detection. The myocardial state index and Golden ratio index are proprietary Harmony device indices, not standard ECG parameters; values reported for exploratory purposes.*

*Parameters with p < 0.05 in within-TMR OR within-Control comparison (paired Wilcoxon signed-rank test). Δ = Post − Pre median. Hedges’ g computed on paired differences (small-sample bias-corrected). * denotes p < 0.05. Primary outcomes marked with *. One TMR baseline recording with an internally inconsistent derived-parameter export (QTcF, Tp–Te, QRS duration, HF-signs index, and the Golden ratio index all implausible despite a normal QT/QTc for the same beat) was excluded from these five derived indices only; the participant's HR, QT, QTc, and all other parameters were retained. Excluding this record left the reported medians and IQR materially unchanged and narrowed the reported ranges accordingly. The Macruz index [P/(PQ−P)] is a ratio that becomes numerically unstable when the P-wave duration approaches the PQ interval; the occasional extreme or negative values (e.g., the negative minimum and large maxima shown in the range) reflect this mathematical instability of the index rather than a physiological measurement and should be interpreted with caution.*

***Table 6.*** *Post-treatment intergroup comparison: TMR vs. Control: HRV parameters*

| **Parameter** | **Control Post (n = 39)** | **TMR Post (n = 35)** | **Δ** | **p** | **Hedges’ g** | **Category** |
| --- | --- | --- | --- | --- | --- | --- |
|  | **median [IQR] (range)** | **median [IQR] (range)** |  |  |  |  |
| SDNN, ms* | 25 [17.5–29.5] (6–55) | 30 [24–38.5] (13–64) | +5 | 0.008 | 0.63 | moderate |
| RMSSD, ms* | 13 [8–16.5] (2–49) | 19 [14–22.5] (3–55) | +6 | 0.001 | 0.62 | moderate |
| SI, units* | 431 [297–890.5] (62–1732) | 253 [179–391] (75–723) | −178 | <0.001 | 0.86 | large |
| HR, bpm* | 80 [72.5–85.5] (49–118) | 69 [65–77] (55–105) | −11 | 0.001 | 0.69 | moderate |
| pNN20, % | 10 [2–14.5] (0–72) | 22 [11–38.5] (0–60) | +12 | <0.001 | 0.83 | large |
| Entropy† | 0.24 [0.07–0.37] (0–0.78) | 0.39 [0.275–0.575] (0.01–0.74) | +0.15 | 0.001 | 0.80 | moderate |
| IVR, units | 671 [543–1264] (152–1992) | 442 [281–707] (140–1274) | −229 | 0.003 | 0.80 | moderate |
| Op. Reg. Ctrl‡ | 60 [51.5–65] (34–80) | 70 [60.5–74] (46–93) | +10 | 0.001 | 0.84 | large |
| Compl. Reg. Idx‡ | 59 [47–67] (33–79) | 69 [62.5–75.5] (35–87) | +10 | <0.001 | 0.82 | large |
| Reg. Reserve‡ | 55 [41–65.5] (22–76) | 67 [59–72.5] (23–82) | +12 | 0.002 | 0.74 | moderate |
| Baevsky FS | 3 [2–5] (0–7) | 2 [1–2] (0–7) | −1 | 0.002 | 0.72 | moderate |

*Post-treatment between-group comparison; five-minute supine recordings. Values are medians with interquartile range [IQR] and (minimum–maximum). Between-group comparison: Mann–Whitney U-test. Effect size: Hedges’ g (unpaired); category per |g| thresholds (0.2 small, 0.5 moderate, 0.8 large). Abbreviations as in Table 2; Baevsky FS, Baevsky functional state score. ‡ Op. Reg. Ctrl (operational regulatory control), Compl. Reg. Idx (complex regulatory index) and Reg. Reserve (regulatory reserve) are proprietary Harmony/OKR indices (0–100), not standard international HRV parameters. † approximate-entropy index; descriptive values are identical to those in Table 4. Table 9 presents a heart-rate-adjusted sensitivity analysis on a smaller complete-case subsample (TMR n = 33, Control n = 34) and its unadjusted p-values and descriptive medians do not exactly match this table.*

** Primary outcomes. Δ = TMR − Control. Positive Δ for HRV; negative Δ for SI/IVR/HR indicate better outcomes in TMR.*

**Table 7.** *Post-treatment intergroup comparison: TMR vs. Control: ECG parameters.*

| **Parameter** | **Control Post (n = 39)** | **TMR Post (n = 35)** | **Δ** | **p** | **Hedges’ g** | **Category** |
| --- | --- | --- | --- | --- | --- | --- |
|  | **median [IQR] (range)** | **median [IQR] (range)** |  |  |  |  |
| HR, bpm | 80 [72.5–85.5] (49–118) | 69 [65–77] (55–105) | −11 | 0.001 | 0.69 | moderate |
| QT interval, s | 0.386 [0.3705–0.4005] (0.338–0.449) | 0.401 [0.3865–0.419] (0.355–0.476) | +0.015 | 0.008 | 0.62 | moderate |
| QTc (Bazett), s | 0.444 [0.4275–0.462] (0.391–0.513) | 0.436 [0.4235–0.447] (0.39–0.496) | −0.008 | 0.144 | 0.30 | small |
| QTcF (Fridericia), s | 0.422 [0.414–0.4325] (0.383–0.481) | 0.427 [0.415–0.433] (0.383–0.482) | +0.005 | 0.665 | 0.05 | negligible |
| Tp–Te interval, s | 0.08 [0.076–0.087] (0.058–0.212) | 0.086 [0.082–0.094] (0.06–0.156) | +0.006 | 0.015 | 0.15 | negligible |
| QRS duration, s | 0.1 [0.095–0.108] (0.066–0.114) | 0.106 [0.096–0.111] (0.058–0.12) | +0.006 | 0.218 | 0.29 | small |
| PQ interval, s | 0.168 [0.154–0.181] (0.118–0.212) | 0.156 [0.14–0.1675] (0.116–0.188) | −0.012 | 0.016 | 0.57 | moderate |
| Macruz index | 1.84 [1.28–2.6] (0.68–49.5) | 1.75 [1.515–2.095] (-17.79–4.27) | −0.09 | 0.935 | 0.48 | small |
| Myocardial state index | 62 [56–72] (42–80) | 63 [55–67] (47–79) | +1 | 0.598 | 0.13 | negligible |
| ST dev. J+80 ms (lead I), mV | 0.045 [0.021–0.072] (-0.030–0.118) | 0.030 [0.016–0.061] (-0.017–0.095) | -0.015 | 0.147 | 0.39 | small |
| Integral ST–T index (aVL) | 69.5 [44.0–85.8] (10.0–100.0) | 50.0 [10.0–83.0] (10.0–100.0) | -19.5 | 0.190 | 0.31 | small |
| K1 = (PQ+QTc)/RR | 0.776 [0.725–0.885] (0.455–0.995) | 0.688 [0.63–0.776] (0.541–1.09) | −0.088 | 0.002 | 0.64 | moderate |
| Golden ratio index | 0.62 [0.485–0.9] (0.06–2.05) | 0.96 [0.76–1.13] (0.16–1.61) | +0.34 | 0.003 | 0.62 | moderate |
| T-symm. by deriv. (aVL) | 0.62 [0.5025–0.73] (0–0.95) | 0.6 [0.13–0.67] (0–0.98) | −0.02 | 0.403 | 0.22 | small |
| T-wave amplitude, lead I, µV | 197 [144–268] (61–337) | 167 [121–245] (46–431) | -30 | 0.243 | 0.19 | negligible |
| T-wave amplitude, lead III, µV | 40 [-44–107] (-125–294) | 47 [-46–128] (-169–224) | +7 | 0.965 | 0.14 | negligible |
| T-wave amplitude, aVL, µV | 88 [36–136] (-72–225) | 79 [0–116] (-52–285) | -9 | 0.353 | 0.10 | negligible |
| Alpha-T angle, frontal, ° | 38 [18–55] (-4–74) | 41 [17–65] (-11–75) | +3 | 0.539 | 0.11 | negligible |

*Post-treatment between-group ECG comparison; five-minute supine recordings. Values are medians with interquartile range [IQR] and (minimum–maximum). Between-group comparison: Mann–Whitney U-test; effect size: Hedges’ g (unpaired); category per |g| thresholds. HR, heart rate; QTc, rate-corrected QT (Bazett); QTcF, rate-corrected QT (Fridericia); Tp–Te, peak-to-end of T wave; ST dev., ST-segment deviation; ISTT, integral ST–T shape index; K1, composite conduction–repolarization index. The myocardial state index and Golden ratio index are proprietary Harmony device indices, not standard ECG parameters; values reported for exploratory purposes.*

** p < 0.05 (Mann–Whitney U test). Δ = TMR Post − Control Post. Effect-size categories: small (≥0.20), moderate (≥0.50), large (≥0.80), very large (≥1.20). ns = not significant. The Macruz index [P/(PQ−P)] becomes numerically unstable when the P-wave duration approaches the PQ interval; the extreme values shown in the range reflect this mathematical instability rather than a physiological measurement, which also explains the discordance between its non-significant rank-based p-value and its parametric effect size. It should be interpreted with caution. The Tp–Te interval likewise contains extreme high-end values (range up to 0.212 s against a median of approximately 0.08 s); because the rank-based Mann–Whitney test is sensitive to a distributional shift whereas Hedges’ g is computed on means and is attenuated by such outliers, a significant p-value may coincide with a negligible effect size for this parameter. The Tp–Te difference should therefore be regarded as statistically detectable but of negligible magnitude.*

**Table 8. Significant HRV–ECG morphological associations in the TMR group at post-treatment visit (FDR q < 0.05; n = 35).**

| **ECG parameter (lead)** | **HRV parameter** | **ρ** | **FDR q** | **Partial ρ*** | **p (partial)** |
| --- | --- | --- | --- | --- | --- |
| Integral ST-T form (II) | pNN50, % | +0.59 | 0.028 | +0.51 | 0.0016 |
| Integral ST-T form (II) | SDSD, ms | +0.60 | 0.028 | +0.53 | 0.0010 |
| T-wave symmetry (max deriv., II) | pNN50, % | -0.57 | 0.028 | -0.52 | 0.0013 |
| ST displacement +0.08 s (II) | Entropy | +0.60 | 0.028 | +0.45 | 0.0071 |
| T-wave symmetry (max deriv., aVF) | pNN50, % | -0.57 | 0.028 | -0.53 | 0.0010 |
| ST displacement +0.08 s (aVF) | Entropy | +0.57 | 0.028 | +0.43 | 0.0106 |
| T-wave symmetry (max deriv., II) | TP, ms² | -0.56 | 0.030 | — | — |
| ST displacement +0.08 s (II) | RMSSD, ms | +0.56 | 0.033 | +0.41 | 0.0146 |
| ST displacement +0.08 s (II) | pNN20, % | +0.53 | 0.037 | — | — |
| ST displacement +0.08 s (II) | pNN50, % | +0.54 | 0.037 | — | — |
| Integral ST-T form (II) | HF, ms² | +0.54 | 0.037 | — | — |
| T-wave symmetry (max deriv., II) | SDSD, ms | -0.54 | 0.037 | — | — |
| T-wave symmetry (max deriv., II) | HF, ms² | -0.53 | 0.037 | — | — |
| ST displacement +0.08 s (aVF) | RMSSD, ms | +0.53 | 0.037 | +0.39 | 0.0210 |
| Integral ST-T form (II) | TP, ms² | +0.53 | 0.037 | — | — |
| Integral ST-T form (II) | RMSSD, ms | +0.53 | 0.037 | — | — |
| ST displacement +0.08 s (aVF) | pNN50, % | +0.52 | 0.039 | — | — |
| ST displacement +0.08 s (II) | SDSD, ms | +0.52 | 0.042 | — | — |
| ST displacement +0.08 s (II) | HF, ms² | +0.51 | 0.050 | — | — |

**Partial ρ: Spearman correlation after residualising both variables on HR. Reported for the three most prominent ECG parameters only. HRV parameters abbreviations: SDNN — standard deviation of NN intervals; RMSSD — root mean square of successive RR differences; SI — Baevsky stress index; pNN50 — proportion of successive RR differences > 50 ms; pNN20 — > 20 ms; SDSD — standard deviation of successive RR differences; HF — high-frequency spectral power (0.15–0.40 Hz); LF — low-frequency spectral power (0.04–0.15 Hz); TP — total spectral power; HR — heart rate. No significant HRV–ST-T morphological associations were detected in the control group or civilian reference group (all FDR q > 0.05).*

**Table 9. Exploratory heart-rate-adjusted sensitivity analysis: post-treatment between-group HRV comparison in the original complete-case subsample (TMR n = 33, Control n = 34; a smaller subsample than the TMR n = 35, Control n = 39 sample used elsewhere in this revision), unadjusted and adjusted for heart rate (rank-based ANCOVA on the five-minute supine recordings).** *After heart-rate adjustment, the between-group differences in SDNN, RMSSD, HF, and approximate entropy (ApEn) were attenuated to non-significance, whereas the Baevsky Stress Index remained significant (p = 0.024) — indicating that most of the between-group HRV advantage is heart-rate-mediated while the reduction in regulatory rigidity is at least partly heart-rate-independent. Values are medians with interquartile ranges (IQR). ApEn, approximate entropy; HF, high-frequency power; LF/HF, low-/high-frequency ratio. p (unadj.): Mann–Whitney U-test; p (HR-adj.): rank-based ANCOVA with heart rate as covariate. Descriptive medians and interquartile ranges are those of the original post-treatment complete-case subsample (TMR n = 33, Control n = 34) and do not exactly match the updated Tables 4 and 6 (TMR n = 35, Control n = 39); this table has not been recomputed on the updated sample pending re-implementation of the rank-based ANCOVA.*

| **Parameter** | **TMR post, median [IQR]** | **Control post, median [IQR]** | **p (unadj.)** | **p (HR-adj.)** |
| --- | --- | --- | --- | --- |
| SDNN, ms | 30 [25–39] | 24 [15–30] | 0.0074 | 0.185 |
| RMSSD, ms | 18.5 [14–24] | 13 [8–17] | 0.0042 | 0.148 |
| Stress Index | 267.5 [178–387] | 437 [294–830] | 0.0007 | 0.024 |
| ApEn | 0.395 [0.25–0.57] | 0.23 [0.08–0.37] | 0.0055 | 0.227 |
| HF, ms² | 112.07 [57.5–190.4] | 46.69 [18.6–116.8] | 0.0077 | 0.152 |
| LF/HF | 2.5 [1.8–3.3] | 3.1 [1.7–3.8] | 0.328 | 0.615 |
| HR, bpm | 69 [66–78] | 79 [71–86] | 0.0075 | covariate |
